# Longitudinal Single‐Cell Imaging of Engineered Strains with Stimulated Raman Scattering to Characterize Heterogeneity in Fatty Acid Production

**DOI:** 10.1002/advs.202206519

**Published:** 2023-06-08

**Authors:** Nathan Tague, Haonan Lin, Jean‐Baptiste Lugagne, Owen M. O'Connor, Deeya Burman, Wilson W. Wong, Ji‐Xin Cheng, Mary J. Dunlop

**Affiliations:** ^1^ Department of Biomedical Engineering Boston University Boston MA 02215 USA; ^2^ Biological Design Center Boston University Boston MA 02215 USA; ^3^ Photonics Center Boston University Boston MA 02215 USA; ^4^ Department of Electrical and Computer Engineering Boston University Boston MA 02215 USA

**Keywords:** longitudinal imaging, metabolic engineering, production heterogeneity, single cell, stimulated Raman scattering

## Abstract

Understanding metabolic heterogeneity is critical for optimizing microbial production of valuable chemicals, but requires tools that can quantify metabolites at the single‐cell level over time. Here, longitudinal hyperspectral stimulated Raman scattering (SRS) chemical imaging is developed to directly visualize free fatty acids in engineered *Escherichia coli* over many cell cycles. Compositional analysis is also developed to estimate the chain length and unsaturation of the fatty acids in living cells. This method reveals substantial heterogeneity in fatty acid production among and within colonies that emerges over the course of many generations. Interestingly, the strains display distinct types of production heterogeneity in an enzyme‐dependent manner. By pairing time‐lapse and SRS imaging, the relationship between growth and production at the single‐cell level are examined. The results demonstrate that cell‐to‐cell production heterogeneity is pervasive and provides a means to link single‐cell and population‐level production.

## Graphical abstract

This study demonstrates longitudinal hyperspectral stimulated Raman scattering (SRS) imaging of chemical production from single metabolically engineered cells. Two advances in SRS microscopy are described, including a method for estimating fatty acid chain lengths and longitudinal imaging of proliferating cells. Using these advances, the study reports discoveries of metabolic heterogeneity among cells and temporal heterogeneity throughout colony formation.

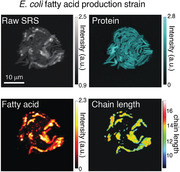

## 1 Introduction

Microbial production of chemicals has the potential to provide a sustainable source of products ranging from fuels to specialty materials.^[^ ^1^, ^2^, ^3^, ^4^ ^]^ A major difficulty holding back the replacement of industrial chemicals with bio‐based alternatives is that bioproduction often falls short in terms of conversion metrics that dictate economic feasibility, such as titer, rate, and yield. Over the past two decades, researchers have made great strides in identifying metabolic pathways capable of producing a diverse array of useful chemicals.^[^ ^5^ ^]^ However, the reality is that extensive engineering and optimization are required for any given chemical to compete as an alternative to those sourced from petroleum.

Producing chemicals in cells offers many advantages, but presents unique industrial challenges. For example, cell‐to‐cell variation and genetic mutations can result in production heterogeneity during fermentation that limits overall process efficiency. Single‐cell variation can stem from a variety of causes, such as stochasticity in the underlying biological processes,^[^ ^6^, ^7^ ^]^ variations in media environments within cultures,^[^ ^8^ ^]^ or selection pressures against high‐producing cells causing mutational escape variants.^[^ ^9^, ^10^ ^]^ However, the frequency and impact of production variation and how it changes over time are largely unknown. Methods that enable quantification of heterogeneity and its emergence are a prerequisite to understanding the root cause and implementing designs that mitigate its effect on overall efficiency.

Here, we focus on fatty acid synthesis, which is an attractive pathway for metabolic engineering because it offers a biological means to synthesize linear hydrocarbons. Fatty acids and their derivatives are high‐demand chemicals that can be used as fuels, commodities, and specialty chemicals. Numerous studies have aimed at increasing the efficiency of fatty acid synthesis pathways as well as controlling the species of fatty acid produced.^[^ ^11^, ^12^, ^13^, ^14^ ^]^ Termination enzymes that interface with this pathway can be used to produce a wide variety of high‐value fatty acids derivatives such as alkanes, olefins, and alcohols.^[^ ^15^ ^]^

Current methods to measure production strain performance include mass spectrometry, fluorescent biosensors, and dyes. Mass spectrometry‐based techniques provide exquisite chemical specificity and, with stable isotope labeling, metabolic fluxes can be inferred.^[^ ^16^ ^]^ However, they are limited in their ability to quantify single cells, which means they can overlook valuable information about population heterogeneity that is key to predicting stability during scale‐up.^[^ ^17^, ^18^, ^19^ ^]^ Further, because the measurement process is destructive, it is not possible to follow production changes within the same cells over time. Biosensor‐based fluorescent assays, in contrast, can capture dynamic, single‐cell information. These systems are amenable to high throughput screens and are non‐destructive.^[^ ^20^ ^]^ However, well‐characterized biosensors are scarce in comparison to the number of chemicals metabolic engineers can produce. Additionally, significant optimization is often necessary to fine‐tune the concentration‐responsive range of a biosensor.^[^ ^21^, ^22^, ^23^ ^]^ In the case of fatty acid production, lipophilic dyes such as Nile red have been used to measure production,^[^ ^24^ ^]^ however these stains lack lipid specificity. Further, both biosensor and dye‐based measurements are indirect readouts of chemical production.

Given the drawbacks of current screening methods, we sought to develop a complementary approach that can capture production and composition information in single cells over time. Stimulated Raman scattering (SRS) is an ideal candidate, as it is a non‐destructive, label‐free vibrational spectroscopic imaging method that directly detects chemical compounds based on intrinsic molecular vibrations.^[^ ^25^, ^26^ ^]^ The ability of SRS to probe metabolic activities in live cells has been demonstrated on microalgae^[^ ^27^ ^]^ and mammalian cells^[^ ^28^ ^]^ for short periods of time. Although SRS imaging of industrially relevant microbes such as *E. coli* has been reported,^[^ ^29^, ^30^ ^]^ its use has been limited to conditions where cells were either fixed or where only a single timepoint was required. Performing longitudinal SRS for compositional chemical imaging on live microbes remains challenging. This is mainly attributed to their small size (e.g., *E. coli* are 1–2 µm in length), which shortens the axial signal integration length, and thus yields weaker SRS signals compared to larger cells. In the context of metabolic engineering, where compositional information on products is critical, one needs to perform hyperspectral SRS to generate pixel‐wise Raman spectra for molecular fingerprinting. However, due to significant spectral overlaps between metabolites, especially in the carbon‐hydrogen (C—H) region, existing hyperspectral SRS image processing methods only provide unsaturation levels of fatty acids.^[^ ^31^ ^]^ They also fail to deliver information on chain length, which is equally important for free fatty acid synthesis.

Here, we introduce a longitudinal hyperspectral SRS method to study metabolically engineered *E. coli*, monitoring free fatty acid production and composition in live cells. We perform SRS in the C—H region which maximizes SRS signals. To overcome spectral crosstalk in the region, we develop a hyperspectral image analysis technique that generates chain length and unsaturation level predictions, allowing for chemical readouts that complement gas chromatograph‐mass spectrometry (GC‐MS) data. First, we demonstrate that we can clearly distinguish fatty acid production strains from wild‐type *E. coli* by deconstructing images into maps of their chemical components. With the ability to measure production at the single‐cell level, we examine heterogeneity in fatty acid production strains and observe both colony‐level heterogeneity and substantial cell‐to‐cell differences in production. We optimize imaging parameters to enable longitudinal hyperspectral SRS imaging to capture fatty acid production over time in growing cells. Next, we use longitudinal measurements to demonstrate dynamic differences in fatty acid production and composition within the same strain. Lastly, we pair SRS microscopy with time‐lapse phase contrast microscopy and automated segmentation analysis to examine relationships between production and growth.

Overall, our study presents two important advances in SRS microscopy, namely fatty acid chain length estimation and longitudinal imaging of proliferating cells. Upon these advances, we characterize metabolic heterogeneity among different cells in a colony and temporal heterogeneity throughout colony formation.

## 2 Results

### 2.1 Hyperspectral SRS Imaging of Fatty Acid Production Strains

**Figure 1:**
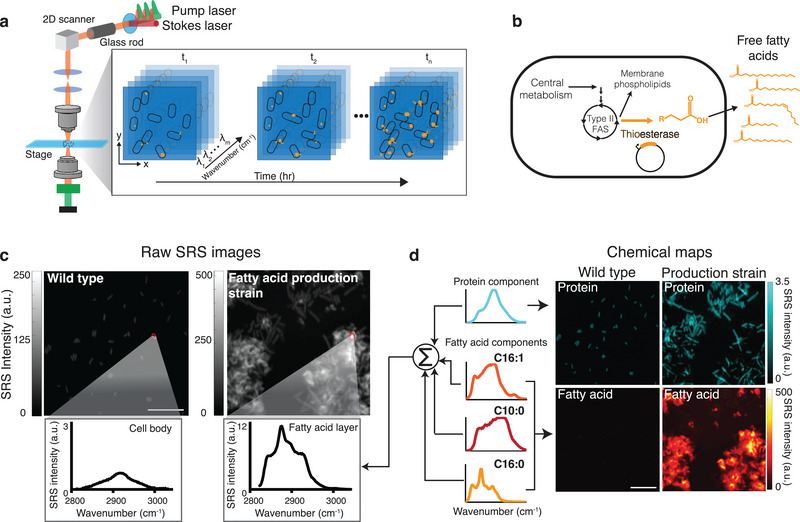
SRS imaging of *E. coli* production strains shows single‐cell free fatty acid levels. a) Schematic of the optical setup for SRS imaging to produce hyperspectral images using a Stokes and pump laser focused on a live sample. Hyperspectral SRS images contain 3D data: *x* and *y* coordinates and wavenumber, which provide spectral information. Longitudinal SRS imaging adds a fourth dimension, time. b) Schematic of free fatty acid production in *E. coli*. Expression of cytosolic thioesterase results in free fatty acid accumulation through the type II fatty acid synthesis (FAS) pathway. Free fatty acids can vary in chain length and unsaturation, largely dictated by thioesterase specificity. c) Representative raw SRS data from wild‐type *E. coli* and a strain overexpressing a cytosolic thioesterase (*Ab*TE*). The summation of Raman spectra at each pixel is shown. Representative regions are outlined in red with the corresponding Raman spectra shown below the image. Fatty acids and proteins emit strong Raman signals in the C—H region (≈2900 cm^−1^). Note that the y‐axis scales are different; Figure S3 (Supporting Information) shows them on the same scale. Scale bar, 10 µm. d) Spectra at each pixel of the SRS image can be decomposed to generate chemical maps. Protein and fatty acid components are decomposed using spectral standards to produce chemical maps. Spectral standards shown in schematic are Bovine serum albumin (cyan), palmitoleic acid (C16:1, orange), capric acid (C10:0, red), and palmitic acid (C16:0, yellow). Protein and fatty acid chemical maps for both strains are shown. Scale bar, 10 µm.

Spectral signals from Raman scattering correspond to vibrational energies of covalent bonds. This allows for direct imaging of chemicals without the need for labels such as fluorescent reporters or dyes. Here, we deploy hyperspectral SRS^[^ ^32^, ^33^, ^34^ ^]^ to obtain chemical maps of protein and fatty acids. To achieve this, we chirp two broadband femtosecond laser beams (pump and Stokes) using high‐dispersion glass rods, producing linear temporal separation of the frequency components (**Figure** **1**a; Figure S1, Supporting Information). The beating frequency of the two beams is linearly correlated with the temporal delay between the two laser pulses. Using a 2D Galvo scanner, the combined laser beam is moved across the *x* and *y* dimensions of the sample to generate an image. This process is then repeated for a range of temporal delays, each of which produces a different wavenumber, ultimately producing a hyperspectral SRS image generated in a frame‐by‐frame manner. The spectral region surrounding the 2900 cm^−1^ wavenumber is typically referred to as the “C—H region” and has a strong SRS signal. Biomolecules such as proteins and fatty acids, which contain many C—H bonds, show a high Raman signal in this region. Importantly, SRS intensity scales linearly with molecular concentrations. The strong signal in the C—H region enables high‐fidelity SRS imaging with low optical powers that are compatible with live‐cell imaging. Thus, this configuration can be used to acquire longitudinal images of live cells, resulting in data across four dimensions: space (*x* and *y*), wavenumber, and time. We set out to utilize SRS chemical imaging in the C—H region to measure fatty acid production in metabolically engineered strains of *E. coli*.

Previous metabolic engineering efforts have focused on producing free fatty acids in *E. coli* using the native type II fatty acid synthesis pathway.^[^ ^14^, ^21^, ^35^ ^]^ Introducing a heterologously expressed acyl‐acyl carrier protein (ACP) thioesterase can catalyze the formation and pooling of free fatty acids from elongating acyl hydrocarbon chains that would otherwise be incorporated into membrane phospholipids (Figure 1b).^[^ ^36^, ^37^ ^]^ We reasoned that SRS imaging could effectively capture fatty acid in production strains since carbon chains present in fatty acids provide strong and distinctive C—H peaks. To test this hypothesis, we studied several production strains that were previously engineered to produce high quantities of free fatty acids (Tables S1 and S2). We first focused on the strain *Ab*TE*, which expresses an acyl‐ACP thioesterase from *Acinetobacter baylyi*, carrying G17R/A165R mutations that improve enzymatic activity in *E. coli*.^[^ ^38^ ^]^ SRS images of *Ab*TE* show increased fatty acid production relative to the wild‐type strain, as evidenced by differences in both the chemical spectra and visible fatty acid droplets around the cells (Figure 1c).

Spatially localized chemical mapping of cell mass and fatty acids can be achieved through linear unmixing of the hyperspectral SRS images. To achieve this, we used a pixel‐wise least absolute shrinkage and selection operator (LASSO) linear unmixing analysis to decompose the hyperspectral image into composite chemical maps of given pure components (Methods). Compared with conventional least‐squares fitting, pixel‐wise LASSO adds a constraint that a few components have dominant contributions at each pixel, which effectively suppresses signal crosstalk between chemical maps. We initially used standard spectra from pure protein (Bovine serum albumin, BSA), saturated fatty acids (C10:0 and C16:0), and unsaturated fatty acids (C16:1) (Figure S2, Supporting Information) to decompose the hyperspectral image into four chemical maps. We selected three types of fatty acids to ensure coverage of fatty acid variation in chain length and unsaturation levels. To visualize total concentrations, all the fatty acid maps were combined to generate a single map. Together with the protein channel, the spectral unmixing procedure outputs two‐channel chemical maps, revealing the distributions of protein and fatty acid components at the single‐cell level (Figure 1d). Protein levels were comparable between wild‐type and *Ab*TE* strains, with slightly elevated levels in the engineered strain. In contrast, the fatty acid signal in the *Ab*TE* strain was significantly stronger than in wild‐type. Wild‐type cells contain membrane phospholipids, which contribute to a small amount of background in the free fatty acid map, however, these signals are much weaker than those recorded in the *Ab*TE* strain (Figure S3a, Supporting Information). This is further validated in the spectral domain, where the average spectrum from wild‐type cells is primarily contributed by protein (Figure S3b, Supporting Information). It should be noted that these strains were sampled from liquid culture, where free fatty acids are secreted and can aggregate in the media. As a consequence, the large fatty acid droplets are not necessarily produced by the cells within the field of view but could be an aggregate of fatty acid produced from many cells in the liquid culture. In subsequent experiments we address this by growing cells on agarose pads to allow for affiliation of cells and the fatty acids they produce, however, these snapshots from liquid culture provide a view into aggregate production.

### 2.2 Characterization of Enzymatic Specificity, Chain Length Distribution, and Degree of Unsaturation

Analytical chemistry methods such as GC‐MS are typically employed to measure chemical production because they offer precise chemical specificity information. For fatty acid quantification, gas chromatography effectively separates fatty acid esters based on chain length and, along with mass/charge spectra, can specifically read out fatty acid ester chain length and unsaturated bonds. From a metabolic engineering perspective, quantification of a fatty acid production strain's chain length distribution and level of unsaturation is critical. For biofuel purposes, chain length and termination chemistry can be tuned to mimic characteristics of fuel sources such as gasoline, diesel, or jet fuel.^[^ ^39^ ^]^ Alternatively, medium‐chain fatty acids (C8‐C12) and their derivatives can be sources of many specialty chemicals.^[^ ^40^ ^]^ With these end point applications in mind, we sought to extend hyperspectral SRS imaging capabilities to capture the specific profiles of free fatty acid production strains.

**Figure 2:**
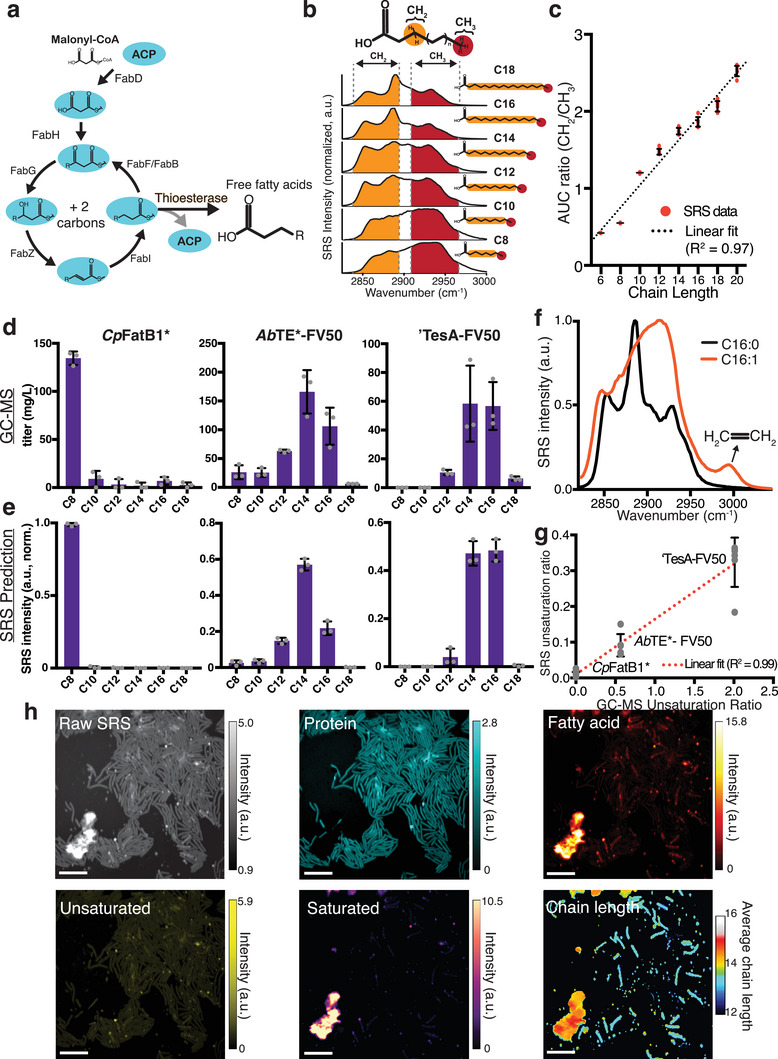
Chain length distribution prediction from different thioesterase enzymes. a) Schematic of the type II fatty acid synthesis pathway in *E. coli*. Introduction of an acyl‐ACP thioesterase pulls out elongating acyl‐ACPs to form free fatty acids. Enzymatic specificity of the thioesterase largely determines the distribution of the fatty acid chain length profile. b) The ratio of internal CH_2_ and terminal CH_3_ bonds within a saturated fatty acid is a function of chain length. Raman spectra of pure saturated fatty acid standards are shown for different chain lengths. Specific spectral windows correspond to each bond. c) The ratio of area under the curve (AUC) of CH_2_/CH_3_ bonds scales approximately linearly with chain length. Error bars show a standard deviation of *n* = 6 replicates. d) Saturated fatty acid chain length distribution prediction with GC‐MS compared to e) SRS using CH_2_/CH_3_ ratio analysis (*n* = 3 biological replicates, error bars show standard deviation). Strains shown are: *Cp*FatB1*, *Ab*TE*‐FV50, and ‘TesA‐FV50 (Table S2, Supporting Information). f) SRS spectra of saturated and unsaturated fatty acid standards (C16:0, C16:1). The unique peak at ≈3000 cm^−1^ allows for spectral decomposition of unsaturation content. g) Comparing GC‐MS unsaturation ratio of produced free fatty acids to SRS production based on spectral analysis. Error bars show standard deviation from n = 5 fields of view for each strain. h) Spectral decomposition and saturated chain length prediction of *Ab*TE*‐FV50 grown on an agarose pad. Scale bars, 10 µm.

In *E. coli*, fatty acid biosynthesis is carried out through a multistep, enzymatic Claisen reduction.^[^ ^41^ ^]^ The enzymatic components of type II fatty acid synthesis in *E. coli* are encoded as separate proteins, creating a pathway in which two carbons are added to an elongating acyl‐ACP chain with each cycle (**Figure** **2**a). The number of cycles around this pathway before the elongating acyl chain is cleaved by an acyl‐ACP thioesterase determines the resulting fatty acid chain length. The primary factor driving chain length is thought to be the enzymatic specificity of the heterologously expressed thioesterase.^[^ ^11^, ^42^ ^]^ Researchers have carried out numerous efforts to engineer specificity of acyl‐ACP thioesterases in order to create desired chain length profiles.^[^ ^14^, ^38^, ^43^, ^44^, ^45^ ^]^ Several thioesterases have been shown previously to produce a range of free fatty acid chain length profiles. Three examples are *Cp*FatB1*, *Ab*TE*, and 'TesA. The CpFatB1* and AbTE* thioesterases originate from *C. palustri*s and *A. baylyi*, respectively, and the “*” denotes mutants that were engineered to increase activity in *E. coli*.^[^ ^38^, ^46^ ^]^ 'TesA is *E. coli*'s native thioesterase, where the “'” denotes deletion of the leader sequence.^[^ ^36^ ^]^ Endogenously, TesA contains a leader sequence that localizes the enzyme to the periplasm; deleting the leader peptide sequence allows for interaction with cytosolic acyl‐ACPs, enabling the production of free fatty acids.^[^ ^36^ ^]^ We worked with strains *Cp*FatB1*, *Ab*TE*‐FV50, and ‘TesA‐FV50, which each express a different thioesterase (Tables S1 and S2, Supporting Information). Strains *Ab*TE*‐FV50 and ‘TesA‐FV50 additionally express heterologous *fadR* and *vhb50*, which have been shown to increase free fatty acid production.^[^ ^12^, ^47^ ^]^ FadR is a transcription factor that regulates many genes in the fatty acid synthesis pathway for increased free fatty acid titer when expressed alongside ‘TesA. Vhb50 is a *Vitreoscilla* hemoglobin that further increases fatty acid production by increasing oxygen uptake.

We conducted an experiment in which each of the three strains was grown in liquid culture and thioesterase expression was induced for 24 h to produce free fatty acids. Samples from each production culture were taken in parallel for GC‐MS quantification and SRS hyperspectral imaging. As expected, GC‐MS results show highly variable chain length distributions depending on the thioesterase expressed (Figure S4, Supporting Information). *Cp*FatB1* primarily produces octanoic acid (C8:0). *Ab*TE*‐FV50 produces a mix of medium‐ and long‐chain saturated fatty acids with myristic acid (C14:0) as the largest component. Lastly, ‘TesA‐FV50 produces long‐chain fatty acids with large contributions from both myristic (C14:0) and palmitic acid (C16:0). Since each production strain has a unique chain length profile, they provide a diverse range of strains for chain length analysis with SRS imaging.

To examine whether different types of fatty acids can be differentiated through SRS spectral features, we acquired SRS spectra of pure free fatty acid standards. We first acquired SRS spectra of various saturated fatty acids, which are inclusive of all that were present in the GC‐MS measurements (C8:0 through C18:0). Importantly, when using pure samples for saturated fatty acids, we noted significant spectral changes with respect to chain length. As shown in Figure 2b, saturated fatty acids in the C—H region are composed of CH_2_ peaks in 2832–2888 cm^−1^ and CH_3_ peaks in 2909–2967 cm^−1^ region.^[^ ^48^ ^]^ Since a saturated fatty acid has an increasing number of CH_2_ bonds as the chain length increases, but the terminal CH_3_ bond number is constant, we reasoned that the ratio of the CH_2_/CH_3_ spectral windows would scale with chain length. With the pure saturated fatty acid standards of variable chain length, we observed a nearly linear (*R* ^2^ = 0.97) relationship between chain length and the ratio of CH_2_/CH_3_ area under the curve (Figure 2c). This suggests that given a mixture of saturated fatty acids, we can utilize the SRS spectra to estimate an average chain length profile.

Meanwhile, unsaturated fatty acids with different chain lengths all have a dominant and broad peak at 2900 cm^−1^, with only small changes in the relative intensities of the 2850 cm^−1^ (CH_2_) and 3000 cm^−1^ (CH_3_) peaks (Figure S5, Supporting Information). The subtle differences between unsaturated fatty acid chain lengths at the CH_2_ and CH_3_ peaks cannot be used to faithfully decompose unsaturated fatty acid chain lengths. Although identifying individual unsaturated fatty acid chain lengths was not possible, we noted that there was a distinct peak at 3000 cm^−1^ that was common to all unsaturated fatty acids (Figure S5, Supporting Information), which allowed us to differentiate whether a fatty acid is unsaturated or saturated. Therefore, in each strain, we used a weighted average of four unsaturated fatty acids (C12:1, C14:1, C16:1, and C18:1) to represent the unsaturated fatty acid channel, in which the weight is derived from relative concentrations of the four unsaturated fatty acids measured by GC‐MS.

We used these relationships to estimate chain length and unsaturation production profiles at the single‐cell level. To obtain these profiles, we selected a total of eight pure chemicals, including protein, averaged unsaturated fatty acid, and a wide range of saturated fatty acids (C8:0, C10:0, C12:0, C14:0, C16:0, and C18:0). We measured spectra for each, normalized them, and applied pixel‐wise LASSO unmixing to decompose the hyperspectral SRS image. We first chose *Ab*TE*‐FV50 as a test dataset for the 8‐component spectral unmixing. However, with an increased number of input references, un‐biased regression became unreliable because of the large number of possible spectral combinations. The resulting signal crosstalk could not be suppressed simply via the sparsity condition in LASSO. To address this issue, we utilized bulk culture strain production data measured by GC‐MS as another physical constraint to augment spectral decomposition. Using strain‐specific GC‐MS quantification in Figure S4 (Supporting Information), we calculated a scaling constant for each fatty acid corresponding to its concentrations and multiplied it with the normalized reference. All types of unsaturated fatty acids were grouped together and yielded a combined scaling constant. This allows the decomposition to start with prior knowledge of a specific production strain to inform the spectral combinations tested for each pixel (Figure S6a, Supporting Information), such that a combination of GC‐MS and SRS measurements can be used together to estimate the average chemical composition per pixel.

Following GC‐MS augmented spectral unmixing, to predict chain length distributions, each saturated chain length chemical map is multiplied by the corresponding spectral reference, summed together, leaving out protein and unsaturated fatty acid maps, to create a hyperspectral SRS image of total saturated fatty acids, which can be used to estimate the average chain length at each pixel using the CH_2_ and CH_3_ spectral windows (Figure S6b,c, Supporting Information). The predicted saturated chain length distributions from SRS samples closely match the features of the GC‐MS distributions in three separate bulk culture samples (Figure 2d,e). We used the Jensen‐Shannon divergence metric^[^ ^49^ ^]^ to quantify similarity between SRS image and GC‐MS distributions, all of which fall under 0.1, representing highly similar distributions (Figure S7, Supporting Information). Importantly, the prediction captures whether the strain produces primarily medium‐ or long‐chain fatty acids or a mixture of both. It is important to note that chain length maps report a single value per pixel even though the pixel may have a mixture of chain lengths. For example, if a mixture of chain lengths is not spatially separated, a non‐even prediction is possible (e.g., 14.9). When comparing predictions with the original GC‐MS data, we bin this as C14 (Experimental Section), however, the complete average chain length values are present in the images. Furthermore, at any pixel, a mixture of fatty acids can be predicted by referencing the individual chemical map outputs for each saturated fatty acid channel (Figure S6b, Supporting Information).

To gauge unsaturation levels, we utilized the presence of the Raman peak at ≈3000 cm^−1^, which is unique to the C = CH_2_ bonds in unsaturated fatty acids (Figure 2f). This peak serves as an identifier of unsaturation level and components from this fatty acid source can be unmixed with LASSO regression. To demonstrate our ability to predict unsaturation levels from production strains, we tested the same three strains, which have different ratios of unsaturation to saturation (Figure S4, Supporting Information). The ratio of unsaturation from GC‐MS data scales linearly with predicted unsaturated ratios from SRS images (Figure 2g), giving an indication of the ability of this approach to predict the ratio of unsaturation. With the ability to calculate unsaturation levels in addition to chain length distributions of saturated fatty acids in SRS images, we cover many aspects of free fatty acid production that are important for metabolic engineers.

We next applied our compositional analysis to *Ab*TE*‐FV50 seeded and grown on agarose pads (Figure 2h). Highly productive strains will secrete end‐products, making it difficult to track the source of produced chemicals back to the cells that generated them. Therefore, sampling from liquid culture for imaging may not accurately provide production heterogeneity information. To increase the likelihood that free fatty acid production is tracked to the cells responsible for production, we first grew cells on agarose pads such that production could be localized to the region containing the cells. We observed a large aggregate of fatty acids outside the cells that are primarily composed of saturated, long‐chain fatty acids. This differs from interpretations of GC‐MS quantification where it is assumed that long‐chain fatty acids remain within the cell.^[^ ^38^ ^]^ Importantly, chain length calculations vary spatially, meaning the analytical augmentation with GC‐MS data is not over‐powering the regression algorithm to produce overly biased predictions. Thus, our results mitigate a potential concern about GC‐MS augmented spectral unmixing, which is that the process will simply output predictions that match the input weights, giving no additional information beyond GC‐MS. Instead, we find chain length predictions from each pixel vary spatially, meaning spectral unmixing is outputting information not solely based on GC‐MS input. Additionally, single‐cell chain length maps display a relatively homogenous makeup of chain lengths between individual cells, which is consistent with current understanding of the fatty acid synthesis pathway and thioesterase specificity.^[^ ^15^ ^]^ However, without single‐cell resolution, it would not be possible to distinguish between this scenario and one where chain‐length mixtures produced from bulk culture originate from distinct subpopulations that produce primarily one chain length each.

### 2.3 Quantification of Heterogeneity in Fatty Acid Production Strains

Given our ability to image production at the single‐cell level, we asked whether our strains displayed production heterogeneity in the overall levels of fatty acid produced. Previous studies have reported sub‐populations within production cultures that are less productive and lead to decreased overall performance of the population in a scaled‐up bioprocess.^[^ ^24^, ^50^ ^]^ Single‐cell chemical imaging with SRS is uniquely suited to quantifying this phenomenon. We focused on strains *Ab*TE*‐FV50 and ‘TesA‐FV50 for agarose pad experiments because *Cp*FatB1* displayed poor growth in the agarose pad conditions.

**Figure 3:**
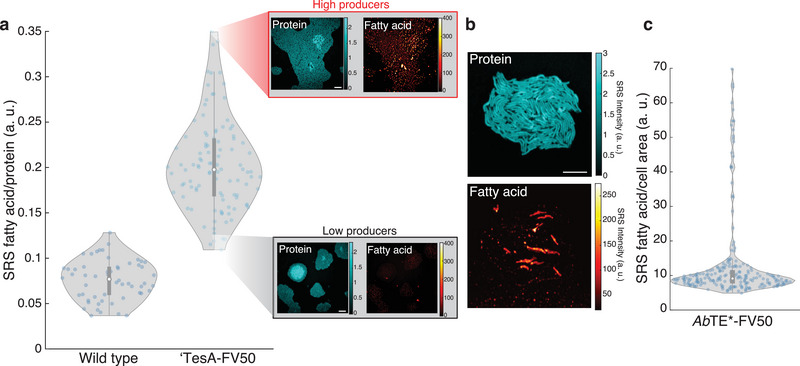
Inter‐ and intra‐colony heterogeneity profiles of production strains. a) Production from replicate ‘TesA‐FV50 microcolonies (*n* = 105) are compared to wild‐type microcolonies (*n* = 56), revealing inter‐colony production heterogeneity. Each data point represents fatty acid production from a single microcolony. Protein and fatty acid chemical maps are shown for representative high and low‐producing microcolonies. Scale bar, 10 µm. b) Representative protein and fatty acid chemical maps are shown for a microcolony of the production strain *Ab*TE*‐FV50. c) Intra‐colony production is quantified for single cells within the microcolony (*n* = 213) (Figure S8, Supporting Information). Each data point represents a single cells’ production. Scale bar, 10 µm. Box plot overlays contain median (white circle), first and third quartiles (gray box), and 1.5× interquartile range (thin gray line) for each distribution.

We first quantified fatty acid production from *E. coli* microcolonies of the wild‐type and ‘TesA‐FV50 production strain (**Figure** **3**a). Interestingly, ‘TesA‐FV50 microcolonies exhibit a high level of colony‐to‐colony production variation. This intercolony heterogeneity is visible in the fatty acid chemical maps, with strains from the same original source exhibiting high and low‐producing microcolonies. One possible explanation for these differences in production is variable transcriptional regulation of key enzymes that are maintained through replication, leading to metabolic bottlenecks.^[^ ^7^, ^51^ ^]^ Alternatively, the ability to manage toxicity associated with production in the time frame following thioesterase induction may lead to divergent production outcomes.^[^ ^52^ ^]^

We also examined production heterogeneity in the fatty acid production strain *Ab*TE*‐FV50. Strikingly, we observed a very different type of production variation in this strain (Figure 3b). Unlike the intercolony heterogeneity in ‘TesA‐FV50, the *Ab*TE*‐FV50 strain has high heterogeneity between cells in a single microcolony. We used the protein channel to segment the image into single cells for analysis (Figure S8, Supporting Information) and quantified single‐cell production (Figure 3c). Our quantification indicates that in this strain a small percentage of cells produce the vast majority of fatty acids. This result is consistent across many fields of view within the microscopy images, suggesting that it is a general feature of this production strain (Figure S9, Supporting Information).

### 2.4 Longitudinal SRS Imaging of Fatty Acid Production During Growth of Colonies

Understanding the dynamics of chemical production with single‐cell resolution can provide key insights into the emergence of heterogeneity, production bottlenecks, and can guide engineering strategies to maximize metabolic flux. To that end, we sought to adapt the SRS system for longitudinal imaging. While SRS imaging of living cells has been reported,^[^ ^27^, ^53^, ^54^ ^]^ its application to chemical production over long periods of growth has not been demonstrated. Previous work from Wakisaka, et al. achieved video rate SRS for short periods of time by reducing spectral acquisitions to four points in the C—H region.^[^ ^27^ ^]^ For metabolic engineering applications, however, spectral fidelity and time scales on the order of bioprocesses would provide a more useful form of longitudinal imaging. Therefore, we sought to develop parameters amenable to longitudinal imaging without loss of spectral information. We installed an incubator on the microscope stage and grew live cells on agarose pads for at least 16 h at 31 °C. First, we tested whether the routine laser powers we used for endpoint SRS imaging were damaging to live cells (75 mW for 1040 nm Stokes and 15 mW for 800 nm pump on sample). At the beginning of longitudinal imaging, we captured a bright field transmission image and measured a hyperspectral SRS image in one field of view (Figure S10a,b, Supporting Information). After 16 h of incubation, cells that were previously exposed to SRS imaging did not duplicate, nor did they produce significant levels of fatty acids. In contrast, cells in a region in the immediate vicinity that had not been exposed to imaging grew into a dense microcolony and produced fatty acid droplets (Figure S10c,d, Supporting Information). Although the laser exposure did not induce visually discernable cell damage, the photodamage altered cell growth, indicating that these laser powers were too high.

To optimize the imaging conditions to reduce phototoxicity, we performed the same live‐cell experiment with lower laser powers. We reduced the Stokes power from 75 to 25 mW, while the pump laser at 800 nm was kept at 15 mW. Interestingly, at the same laser scanning conditions as previous experiments, namely 150 nm pixel step size and 10 µs pixel dwell time (Figure S11a, Supporting Information), cells were alive and maintained metabolic activities, as fatty acid content showed up inside cells. However, fatty acids did not form droplets as shown in single‐shot SRS experiments (e.g., blue box region in Figure S10, Supporting Information), indicating cell function perturbation can occur at lower powers than those altering cell growth. To lower laser exposure further, we increased the step size of each laser scan from 150 nm to 230 nm at a fixed dwell time of 10 µs, corresponding to a shorter laser exposure per unit area (Figure S11b, Supporting Information). Cells under this condition showed no obvious metabolic activity perturbation, as they continued to grow, produced fatty acids, and formed droplets. As an additional check that these imaging conditions did not have a deleterious impact on cells, we utilized a stress‐responsive promoter, P_ibpAB_, to control expression of the fluorescent reporter mRFP1 (Figure S11c, Supporting Information). P_ibpAB_ is driven by the heat shock *σ*‐factor (*σ* ^32^) and is upregulated in response to stress.^[^ ^55^ ^]^ We exposed cells to the 25 mW/15 mW laser intensities at 150 nm and 230 nm step sizes, respectively, obtained co‐registered wide‐field fluorescence images (Figure S11c, Supporting Information), and compared promoter activity to cells that received no SRS exposure. In the 150 nm condition, mRFP1 expression indicates that intracellular stress was significantly upregulated in response to SRS exposure. With the increased step size of 230 nm, mRFP1 expression was equivalent to that from the cells that received no laser exposure (Figure S11d, Supporting Information). To rule out the possibility that a few cells were growing well while others died which could be obscured in dense cultures, we sparsely seeded cells and imaged growth. With the optimized conditions of 25 mW/15 mW laser intensities and 230 nm step size, we measured transmission and SRS images for the same field of view of sparsely seeded cells after 3 and 5 h of incubation, seeing clear evidence of normal growth even after SRS imaging in all single cells (Figure S12, Supporting Information). We took a final wide‐field image at 6 h, which showed that cells continued to replicate normally. Therefore, we concluded that by both reducing laser powers and increasing step size, we established a condition that allows for longitudinal SRS imaging of *E. coli*.

**Figure 4:**
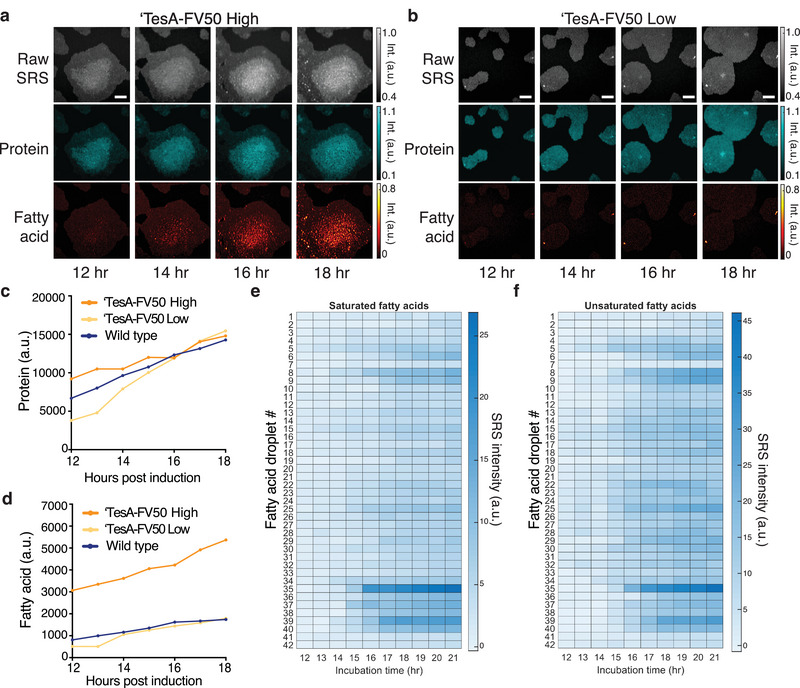
Longitudinal SRS imaging of production dynamics. Time‐lapse images of a) a ‘TesA‐FV50 high‐producing microcolony and b) a ‘TesA‐FV50 low‐producing microcolony under the same conditions, shown with the raw SRS images (spectral summation of the SRS image stack) and chemical maps corresponding to protein and fatty acid content. Scale bars, 10 µm. Quantification of c) protein and d) fatty acid over time from the microcolonies in (a,b) and Figure S13, Supporting Information). e) Saturated and f) unsaturation content of individual droplets from the ‘TesA‐FV50 high microcolony shown in (a). Locations for all numbered droplets are shown in Movie S5 (Supporting Information).

With these optimized imaging conditions, we first tracked fatty acid production within the strain ‘TesA‐FV50. In line with theheterogeneity patterns we originally observed in this strain (Figure 3a), the production trajectories varied across microcolonies (**Figure** **4**a,b). In one example, fatty acid signals increased in cells starting ≈12 h after thioesterase induction (Figure 4a). After the microcolony reached a high cell density on the agarose pad, we observed a significant accumulation of fatty acids. In contrast, a second microcolony of the same strain produced only low levels of fatty acid (Figure 4b). For comparison, we also tracked the growth and fatty acid production of wild‐type cells under the same conditions, observing only low levels of fatty acid production (Figure S13, Supporting Information). Time‐lapse wide‐field transmission images for the wild‐type strain show that cells under SRS laser exposure grew well during the entire experiment period and at levels comparable to those regions not exposed to imaging, reaffirming that these conditions are non‐toxic (Movie S1, Supporting Information). We quantified fatty acid and protein levels of each microcolony and the wild‐type strain. Protein levels in each strain increased at comparable rates (Figure 4c). Fatty acid levels in the wild‐type colony increased modestly while the high‐producing ‘TesA‐FV50 microcolony fatty acid levels increased dramatically (Figure 4d). The low‐producing ‘TesA‐FV50 microcolony produced fatty acids at levels comparable to wild‐type.

The activity in the high‐producing ‘TesA‐FV50 microcolony is in line with known regulation patterns in *E. coli* fatty acid synthesis. When high cell density is reached in wild‐type *E. coli*, the pathway is inhibited by a buildup of acyl‐ACPs. This mechanism is reported to act through direct inhibitory interactions with key enzymes within the pathway, such as acetyl‐CoA carboxylase, FabH, and FabI.^[^ ^56^, ^57^ ^]^ Additionally, acyl‐ACP or acyl‐CoA responsive transcription factors, FadR and FabR, respectively, act to regulate transcriptional responses that control fatty acid synthesis.^[^ ^58^, ^59^ ^]^ In the presence of a cytosolic thioesterase, as in the ‘TesA‐FV50 strain, this inhibition is released through the conversion of accumulated acyl‐ACPs to free fatty acids. However, thioesterase expression is induced starting at t = 0 h, and significant accumulation of fatty acid does not happen until the microcolony is well established. Even with the ‘TesA thioesterase highly expressed, phospholipid metabolism may dominate metabolic flux through the fatty acid synthesis pathway until sufficient density is reached to suppress incorporation of acyl‐ACPs into phospholipids. A recent study from Noga et al. uncovered a post‐translational mechanism that modulates phospholipid biosynthesis through PlsB acyltransferase and ppGpp, which may explain the delay in free fatty acid accumulation.^[^ ^60^ ^]^ This regulation, along with upstream feedback regulation of fatty acid synthesis, may be involved in the microcolony production phenotypes.^[^ ^57^ ^]^ However, we note that only free fatty acids are included in our GC‐MS fatty acid measurements.

Additionally, we measured the dynamics of the *Ab*TE*‐FV50 fatty acid production strain, which produces a variety of medium‐ and long‐chain fatty acids (Figure S4, Supporting Information), with significant heterogeneity in production among cells (Figure 3b,c). We again observed fatty acid production over time, with similar delays in fatty acid accumulation despite thioesterase induction at *t* = 0 h (Figure S14a, Supporting Information). In this strain, a few cells within the microcolony produce large amounts of fatty acid. The production dynamics for these few cells are similar to fatty acid production within the ‘TesA‐FV50 strain, but the remainder of cells exhibit low levels of production for the duration of imaging.

To further understand the dynamics of fatty acid production, we tracked the composition of individual droplets from the high‐producing ‘TesA‐FV50 microcolony and high‐producing cells from the *Ab*TE*‐FV50 microcolony. Both saturated and unsaturated fatty acid levels increase similarly within the droplets of the ‘TesA‐FV50 strain (Figure 4e,f). Interestingly, the high‐producing cells from the *Ab*TE*‐FV50 strain initially produce saturated fatty acids, but saturated fatty acid levels plateau in a subset of cells as the incubation continues (Figure S14b,c, Supporting Information). In contrast, unsaturated fatty acid production continues to increase for the duration of the experiment (Figure S14d,e, Supporting Information). Additionally, we analyzed the chain length composition for both strains longitudinally (Figure S15a,b, Supporting Information). Droplets from ‘TesA‐FV50 were primarily C14 on average. Chain lengths for *Ab*TE*‐FV50 high producer cells displayed high fluctuations at earlier time points but gradually converged to the range of C12 – C14. We believe the early fluctuations stem from a decreased signal‐to‐noise ratio at low fatty acid concentrations, especially under the low power conditions needed for longitudinal imaging. When the signal‐to‐noise ratio is increased for stronger SRS signals, such as for the large extracellular droplets at later time points, the chain length prediction becomes more reliable and stabilizes to a range between C12 – C14.

### 2.5 Single Cell Growth‐Production Relationship

**Figure 5:**
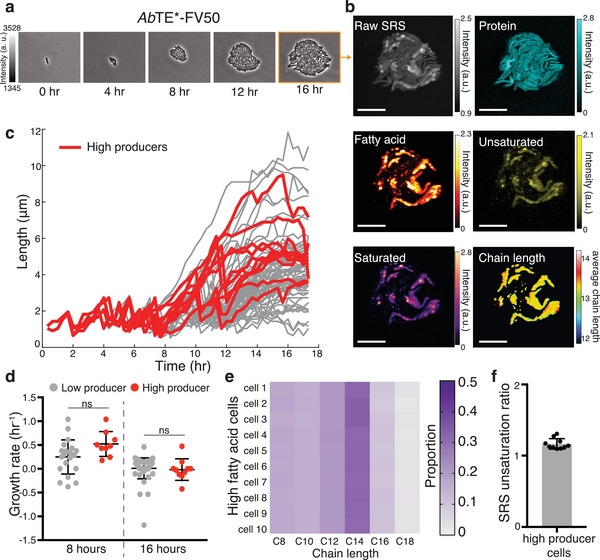
Single‐cell growth‐production relationship. a) Time‐lapse phase contrast imaging of an *Ab*TE*‐FV50 microcolony followed by b) endpoint SRS imaging and spectral decomposition. Scale bars, 10 µm. c) Single‐cell lengths as a function of time within the microcolony shown in (a,b), with high producer trajectories highlighted in red (*n* = 68 cells total). Sharp decreases in length correspond to cell division events. High producers are defined as the top 15% of producer cells (Figure S18, Supporting Information). d) Growth rate comparisons of high and low producer trajectories at 8 and 16 h (*p* = 0.0507 and *p* = 0.714, respectively; two‐tailed unpaired t‐test). Growth rate is calculated from cell length data in (c) (Experimental Section). e) Average saturated chain length prediction of high producer cells. Total saturated fatty acid amount is normalized to 1. f) Unsaturation ratio (unsaturated/saturated) of high producer cells. Error bars show standard deviation.

Next, we asked whether cell‐to‐cell differences in fatty acid production correlate with differences in growth rates between cells. Production of a heterologous product is often associated with changes in cell physiology due to the consumption of resources and intermediate or end‐product‐associated toxicities.^[^ ^61^, ^62^, ^63^ ^]^ Consequently, we asked whether growth rate is inversely correlated with fatty acid production. For this analysis, we focused on the *Ab*TE*‐FV50 strain because it exhibits significant intracolony heterogeneity. At the bulk culture level, we do not observe a decrease in growth when production is induced through *Ab*TE*‐FV50 expression (Figure S16, Supporting Information). However, bulk culture measurements do not rule out slow growth of a high‐producing subpopulation. To understand whether there exists a growth tradeoff in the high‐producer subpopulation, we measured growth at the single‐cell level. Although we can resolve single cells using the longitudinal SRS conditions, the lowered resolution needed to avoid phototoxicity hinders single‐cell segmentation to quantitatively probe growth at many time points. To avoid these limitations, we used a combination of time‐lapse, phase contrast microscopy followed by endpoint SRS imaging (**Figure** **5**a). Using the high‐resolution phase contrast images, we then segmented and quantified single‐cell growth rates using an automated segmentation pipeline for microcolonies.^[^ ^64^ ^]^ Pairing growth quantification with endpoint SRS, we tracked the growth trajectories and lineages of single cells within the microcolony to their fatty acid production. Spectral decomposition of the endpoint SRS image allows the high fatty acid cells to be identified, along with other chemical composition information (Figure 5b). In addition, because we use a single endpoint measurement, we returned to the original imaging conditions (15 mW pump, 75 mW Stokes, 150 nm step size), allowing us to increase the spectral signal‐to‐noise ratio. Growth of the high‐producer cells in the microcolony, measured as cell length over time, did not vary substantially from low‐producer cells (Figure 5c, Figure S17, Movies S2–S4, Supporting Information). We binned cells into two groups, low and high fatty acid producers, where we defined high producers as those with production in the top 15% of single cells in the distribution (Figure S18, Supporting Information). Examining the growth rates of each cell near the endpoint (16 h) and earlier in the time course (8 h) shows that, in both cases, growth rate is not significantly different between the high and low producers.

Given our ability to decompose the fatty acid signal into unsaturated and chain‐length components, we also analyzed the top producer cells’ composition to gain further insight into the high fatty acid phenotype in this strain. We found that high‐producer cells have similar chain length distributions between cells. In line with GC‐MS measurements sampled from bulk culture (Figure 2d), each cell contains a wide distribution of chain lengths with C14:0 being the most dominant (Figure 5e). However, single‐cell distributions are enriched in medium‐chain fatty acids, especially C8:0 and C10:0, and have comparably low levels of C16:0 relative to GC‐MS measurements of bulk culture. The single‐cell chain length predictions result from saturated fatty acid map outputs (Figure S6b, Supporting Information), which are able to predict mixtures of fatty acids at each pixel. Additionally, relative to bulk culture sampling, the unsaturation ratio of the top producers is significantly increased in high‐producer cells (Figures 5f and 2g). The chain length trend remains consistent when the threshold for high‐producer cells is decreased (Figure S19, Supporting Information). The decreased levels of C16 present in the high fatty acid cells relative to bulk culture may be related to unsaturated fatty acid biosynthesis. In *E. coli* fatty acid synthesis, double bonds in the carbon tail of elongating fatty acids are formed specifically when the carbon chain has reached decanoyl‐ACP (C10), followed by further elongation to C12:1, C14:1, or C16:1.^[^ ^65^ ^]^ It is possible that chain lengths that would have otherwise reached C14:0 and C16:0 are instead unsaturated and result in the shorter‐than‐expected average chain length predictions. These differences may be the result of longer‐chain fatty acids being produced in the numerous low‐producer cells at comparatively low levels, differences in culturing conditions, or other factors.

## 3 Discussion

Chemical imaging can play a key role in the strain engineering process. Current quantification techniques rely either on methods like GC‐MS, which are chemically‐specific but where information about individual cells and their dynamics are lost, or on fluorescent reporters or dyes, which are indirect readouts and can be difficult to engineer or limited in their specificity. SRS imaging has the potential to dramatically improve this process by providing key insights into chemical production at the single‐cell level. Thus, methods that were previously only accessible with single‐cell readouts, such as directed evolution or cell‐sorting approaches are in principle possible with SRS imaging. Further, the ability to track production changes over time can provide insight into the emergence of production heterogeneity and, ultimately, guide strategies to avoid low producers in the population. The landscape for strain engineering is expanding rapidly, with systems biology approaches to enzyme engineering and novel technologies for quantifying production offering great promise for improving designs. In this study, we focus on fatty acid synthesis, which is an important pathway that can be engineered to produce a diversity of valuable chemicals. Development of this pathway toward near theoretical yields will be important to replace many industrial chemicals with sustainable bio‐based alternatives.^[^ ^5^ ^]^

Here, we examined free fatty acid production strains of *E. coli* using SRS and demonstrated that hyperspectral imaging allows for image decomposition into major chemical components, with the ability to distinguish cells from their chemical product. By incorporating additional analysis, we also introduce an approach that can estimate chain length distribution and unsaturation degree, increasing the amount of information that can be extracted from SRS hyperspectral images. These advances can enable a metabolic engineer to examine fatty acid production strains using SRS imaging while maintaining chemical specificity data.

Visualizing chemical production at the single‐cell level reveals important information that would otherwise be obscured by bulk culture quantification methods. We demonstrate this by examining production heterogeneity among different engineered strains, observing both intra‐ and inter‐colony differences in production within microcolonies. These results provoke fundamental questions about the mechanisms leading to cellular heterogeneity and also suggest that engineering strategies that eliminate low‐producers could improve yields. For example, it may be possible to gradually enhance the overall production levels of a strain of engineered *E. coli* through multiple cycles of growth and dilution, with a step that removes low‐producers at the end of each cycle.

Furthermore, we established parameters that allow us to extend SRS imaging for longitudinal studies in live cells. Unlike previous phototoxicity studies focusing on acute responses like membrane blebbing,^[^ ^66^, ^67^ ^]^ we directly observe long‐term cell functions including cell replication, free fatty acid synthesis, and the absence of induction of stress response. SRS imaging has been used to probe metabolism in live cells previously, such as in studies by Wakisaka et al.^[^ ^27^ ^]^ and Ota et al.,^[^ ^68^ ^]^ and we extend these results in several critical ways. In our experiments, we track the same cells over multiple hours, rather than sampling new cells from liquid culture at each timepoint as in Wakisaka et al. In Ota et al., the authors study the same cell over time using Raman and SRS, however only at two timepoints, instead of multiple timepoints throughout. In addition, we use *E. coli* for our study while Wakisaka et al. and Ota et al. use the alga *Euglena gracilis*. *E. coli* are highly amenable to metabolic engineering, but their small size makes both imaging and analysis more challenging (*E. coli* are 1–2 µm in length while *E. gracillis* are 35–50 µm^[^ ^69^ ^]^). Thus, our results significantly extend prior findings, offering longitudinal imaging of a highly relevant engineered species. We envision production tracking at the single‐cell level will be valuable for metabolic engineering studies by establishing how and when heterogeneity emerges. To quantify single‐cell properties such as growth rate, however, higher resolution longitudinal imaging is needed to achieve time‐lapse data that can be processed with segmentation algorithms. Further development focused on mitigating phototoxicity without decreasing resolution may be able to overcome this challenge in the future.

As we demonstrate, a hybrid approach using phase contrast imaging and endpoint SRS microscopy allows for fundamental questions to be examined, such as the growth‐production tradeoff. Interestingly, in the *Ab*TE*‐FV50 strain that we studied using this hybrid approach, we observed no tradeoff between growth and production. This information, along with insights into the composition of the high fatty acid cells, can lead to novel hypotheses of the underlying cause of intracolony heterogeneity in this strain. It should be noted that, given the overlap between cell body and fatty acid signal, we interpret some cells to be high producers, however, there is a possibility that these fatty acids originate from other cells and aggregate, and increased temporal resolution of imaging could help to clarify this in future experiments. Nevertheless, our results underpin the utility of examining single‐cell characteristics to increase performance of a given strain. For example, recent approaches to increase bioproduction involving dynamic regulation, either through transcriptional feedback circuits or optogenetic regulation, show promise to increase strain efficiency.^[^ ^70^, ^71^ ^]^ Imaging single‐cell production dynamics in these strains could increase our understanding of how feedback systems can be used in the context of metabolic engineering. Together with synthetic biology methods, our system has the potential to answer fundamental questions relating to the production of biosynthetic targets at the single‐cell level. Further, because SRS imaging does not require engineered biosensors, it has the potential to serve as a widely useful platform to boost the pace of strain engineering for a broad range of metabolites.

Moving forward, it will be important to understand the connection between production at the single‐cell level and bulk culture output. Imaging fields of view sampled from bulk culture can potentially lead to biased overall titer prediction, especially if the product is not soluble in water. Alternatively, studying microcolonies grown on agarose pads is ideal for imaging but not necessarily predictive of bulk culture behaviors. For example, nutrient mixing, population selection, and secretion may differ between the 2D growth conditions and a well‐stirred liquid culture. Additionally, SRS has sensitivity limits significantly higher than mass spectrometry^[^ ^72^ ^]^ and thus requires a product to be produced in sufficient quantities before SRS can be used to guide further engineering. Given these limitations, we envision that SRS studies will be most useful for strain optimization rather than enzyme or pathway discovery.

SRS imaging in different spectral regions, such as the fingerprint region (400–1800 cm^−1^), can be adapted to study strains producing non‐fatty acid‐derived chemicals of interest, such as terpenes, to expand the scope of SRS imaging in metabolic engineering.^[^ ^30^ ^]^ In addition, because the approach is label‐free it does not require biosensors with fluorescent reporter readouts, making it amenable to quantification of production in organisms that are recalcitrant to genetic modification. Moreover, instrumentation advances can enable SRS‐guided single‐cell screening, such as SRS‐based cell sorting, which has been demonstrated recently for cell phenotyping.^[^ ^73^ ^]^ The throughput we achieve in this study is limited by spectral tuning of the motorized delay stage and time spent manually focusing on samples. In future work, applying the ultrafast spectral tuning SRS system from Lin et al.,^[^ ^30^ ^]^ along with integrated autofocusing could drastically increase throughput. Much like the utility of fluorescence‐activated cell sorting in synthetic biology applications, we envision that SRS‐based cell sorting could increase the throughput of strain screening and enable directed evolution based on chemical production in the future. This work acts as a jumping‐off point for SRS imaging in metabolic engineering to aid in the development of more efficient strains for renewable chemical production.

## 4 Experimental Section

### Bacterial Strains and Plasmids

Plasmid and strain information are listed in Tables S1 and S2 (Supporting Information). The pBbA5c‐‘tesA‐vhb50‐8fadR plasmid was a gift from Dr. Fuzhong Zhang. The BW25113 Δ*fadE* strain was from the Keio collection,^[^ ^74^ ^]^ and the FLP recombination protocol from Datsenko and Wanner was used to cure the *kan^R^* cassette from the genome.^[^ ^75^ ^]^ Golden gate cloning was used^[^ ^76^ ^]^ to create the pBbA5c‐vhb50‐8fadR plasmid by deleting the coding sequence of ‘*tesA* from pBbA5c‐‘tesA‐vhb50‐8fadR. The pBbA5c‐CpFatB1.2‐M4‐287 plasmid was also constructed using golden gate cloning, with the pBbA5c backbone amplified from the BglBrick plasmid library^[^ ^77^ ^]^ and the coding sequence of *Cp*FatB1.2‐M4‐287 derived from Hernández Lozada et al.^[^ ^46^ ^]^ and synthetized by Twist Biosciences (South San Francisco, CA). pSS200 was a gift from Dr. Pamela Peralta‐Yahya. pBbE‐ibpAB‐mRFP1 was constructed using the pBbE5k BglBrick backbone^[^ ^77^ ^]^ with the promoter region of the genomic *ibpAB* operon as in Ceroni et al.^[^ ^55^ ^]^ pBbA5c‐‘tesA‐sfGFP‐vhb50‐8fadR and pSS200‐sfGFP were constructed using golden gate cloning with pBbA5c‐‘tesA‐vhb50‐8fadR and pSS200 as backbones, respectively, along with an sfGFP coding sequence containing a flexible GS linker to insert in frame with each thioesterase.

### Growth and Induction of Fatty Acid Production Strains

For fatty acid production experiments, pre‐cultures were grown overnight in LB media and used to inoculate 3 mL M9 minimal media (M9 salts, 2 mM MgSO_4_, 100 µM CaCl_2_) with 2% glucose and grown at 37 °C with 200 rpm shaking. Antibiotics were added to the media where necessary for plasmid maintenance according to resistances in Table S1 (Supporting Information) (100 µg mL^−1^ for carbenicillin and 25 µg mL^−1^ for chloramphenicol). The cultures were allowed to grow until approximately OD_600_ = 0.6 before thioesterase expression was induced with IPTG. Induction levels were 500 µM for ‘TesA‐FV50 and 50 µM for *Ab*TE*, *Ab*TE*‐FV50, and *Cp*FatB1*. For imaging from liquid cultures, cells were grown for 24 h after IPTG induction and then 3 µL of sample was taken for imaging. Samples from liquid culture were placed on 3% agarose pads (Promega) containing M9 minimal media and sandwiched between glass coverslips to immobilize the cells for imaging. Samples from liquid culture were allowed to dry on the agarose pads for ≈15 min prior to imaging. For longitudinal imaging, production heterogeneity experiments, and phase contrast imaging, once cells reached OD_600_ = 0.6 in liquid culture, the sample was placed on a 3% low melting point agarose pad containing M9 minimal media with 2% glucose, IPTG as specified above, and appropriate antibiotics for plasmid maintenance, as detailed in Table S1 (Supporting Information). Microcolonies were imaged after 18 h of growth on the agarose pads at 31 °C.

For the chain length distribution prediction, cultures were induced with IPTG in liquid cultures for 24 h. At the 24 h timepoint, 3 µL of sample was taken for imaging, and another sample of the culture was taken for GC‐MS analysis to allow a direct comparison of the same culture. Five fields of view were imaged for each culture.

### Fatty Acid Derivatization and Quantification with GC‐MS

Samples for GC‐MS quantification were taken 24 h post‐IPTG induction. 400 µL of vortexed culture was taken for fatty acid extraction and derivatization into fatty acid methyl esters as described by Sarria et al.^[^ ^38^ ^]^ with the following minor modifications: Internal standards of nonanoic acid (C9) and pentadecanoic acid (C15) were added to the 400 µL sample at final concentrations of 88.8 mg L^−1^ each and vortexed for 5 s. The following was then added to the sample for fatty acid extraction and vortexed for 30 s: 50 µL 10% NaCl, 50 µL glacial acetic acid, and 200 µL ethyl acetate. The sample was then centrifuged at 12 000 g for 10 mins. After centrifugation, 100 µL of the ethyl acetate layer was mixed with 900 µL of a 30:1 mixture of methanol:HCl (12N) in a 2 mL microcentrifuge tube. The solution was vortexed for 30 s followed by incubation at 50 °C for 60 min for methyl ester derivatization. Once cooled to room temperature, 500 µL hexanes, and 500 µL water were added to the 2 mL microcentrifuge tube, vortexed for 10 s, and allowed to settle. 250 µL of the hexane layer was mixed with 250 µL ethyl acetate in a GC‐MS vial for quantification.

The samples were analyzed with an Agilent 6890N/Agilent 5973 MS detector using a DB‐5MS column. The inlet temperature was set to 300 °C with flow at 4 mL min^−1^. The oven heating program was initially set to 70 °C for 1 min, followed by a ramp to 290 °C at 30 °C min^−1^, and a final hold at 290 °C for 1 min. GLC‐20 and GLC‐30 FAME standard mixes (Sigma) were tested using this protocol to ensure proper capture of all chain lengths and to gauge retention times. Internal standards were used for quantification, with chain lengths C8‐C12 quantified with the nonanoic acid internal standard and C14‐C18 quantified with the pentadecanoic internal standard.

### Optical Setup

The SRS setup was driven by an 80 MHz femtosecond laser (Insight Deepsee+, Spectra‐Physics, USA) with two synchronized outputs. One output was fixed at 1040 nm with a pulse duration of ≈150 fs, while the other was tunable from 680 – 1300 nm with ≈120 fs pulse width. The 1040 nm beam was used as the Stokes and was modulated by an acousto‐optical modulator (522c, Isomet, USA) at 2.5 MHz. The tunable output was set to 798 nm to excite the C—H region and spatially combined with the Stokes by a dichroic mirror. Six 15 cm SF‐57 glass rods were used to linearly chirp the femtosecond pulses to ≈2 ps. Five of the rods were placed on the common path while one was placed on the Stokes path to parallelize the degree of chirping considering its longer wavelength. A motorized delay stage was used to scan the temporal delay between two pulses to tune the excitation frequency. The combined beams were sent to a pair of 2D Galvo scanners (GVSM002, Thorlabs, USA) to perform laser scanning imaging. A 40× oil‐immersion objective was used (RMS40X‐PFO, Olympus, Japan) to focus the laser onto the sample. Powers on the sample were 15 mW for pump and 75 mW for Stokes, with a pixel step size of 150 nm and 10 µs pixel dwell time. For longitudinal imaging experiments, the Stokes power was reduced to 25 mW, with a pixel step size of 230 nm and 10 µs pixel dwell time. A home‐built resonant amplifier photodiode collects and amplifies the stimulated Raman loss signal at the modulation frequency. A lock‐in amplifier was used (UHFLI, Zurich Instruments, Switzerland) to extract the signal and send it to a data collection card (PCIe‐6363, National Instruments, USA). It was noted that all elements described here were commercially available with the exception of the photodiode, which had been previously reported.^[^ ^78^ ^]^ Custom LabView (National Instruments, USA) software was used to synchronize the Galvo scan with the delay line scan to obtain a hyperspectral SRS image stack in a frame‐by‐frame manner.

### Pixel‐Wise LASSO for Hyperspectral Image Unmixing

To obtain concentration maps for chemicals, linear unmixing was performed on the raw hyperspectral image stack. Assuming the number of pure components as *K* and the dimensions of a hyperspectral image as *N_x_*, *N_y_*,*N*_*λ*_, the unmixing model can be written as:

(1) D=CS+E

where $D\in R^{N_{x}N_{y}\times N_{\lambda }}$ is the raw data reshaped as a 2D matrix in raster order, $C\in R^{N_{x}N_{y}\times K}$ is the collection of concentration maps, $S\in R^{K\times N_{\lambda }}$ contains SRS spectra of all the components, while *E* is the residual term with error and noise. Given the prior knowledge of spectra for all the pure components, the task was reduced to generating chemical maps *C* via least square fitting. To avoid crosstalk between spectrally overlapped components, a *L*1 norm sparsity constraint was added by observing that at each spatial position, a few components dominate the contribution. The solution for *C* was found in a pixel‐by‐pixel manner by solving for the following optimization problem known as the least absolute shrinkage and selection operator (LASSO):

(2) $${\hat{C}}_{i}=\arg\;\min_{C_{i}}\left\{\frac{1}{2}\;{\left\|D\left(i,:\right)-C_{i}S\right\|}^{2}+\beta {\left\|C_{i}\right\|}_{1}\right\}$$

where *i* represents a specific pixel in the hyperspectral image, ${\hat{C}}_{i}$ stands for the estimated concentrations for all components at pixel *i*, and *β* is a hyperparameter controlling the level of *L*1 norm regularization at each pixel.

### GC‐MS Augmented Single‐Cell Fatty Acid Composition Analysis

To obtain the compositional information of fatty acids, pure fatty acid references were measured first based on strain‐specific GC‐MS measurements of fatty acid composition. Specifically, bovine serum albumin (BSA) was used as the protein standard; C8:0, C10:0, C12:0, C14:0, C16:0, and C18:0 as the saturated fatty acids standards; and C12:1, C14:1, C16:1, and C18:1 as the unsaturated fatty acids standards. All standards were sourced from Sigma Aldrich, USA. Due to the spectral similarities between unsaturated fatty acids, unsaturated fatty acid chain length could not be differentiated, and thus used a single unsaturated fatty acid derived from a weighted average of normalized spectra of all four unsaturated fatty acids. The weights were derived from GC‐MS measurements to reflect relative percentage of each component. For saturated fatty acids, the same approach to rescale normalized spectra to reflect relative abundance was used. With a total of eight input references, pixel‐wise LASSO unmixing was run to generate eight chemical concentration maps.

To obtain average chain length information for the saturated fatty acids, a hyperspectral, saturated fatty acid image was created by summing over the product of concentration and reference for all the saturated fatty acids (Figure S6b, Supporting Information). The area under the curve ratio of CH_2_ to CH_3_ was then calculated for each pixel, using 2832 to 2888 cm^−1^ for CH_2_ and 2909 to 2967 cm^−1^ for CH_3_ (Figure S6c, Supporting Information). The linear relationship of ratio to chain length produced from standards was used (C6:0 – C20:0, Sigma Aldrich, USA) to calculate a predicted average chain length for each pixel. For single‐cell chain length distributions (Figure 5e; Figure S19, Supporting Information), the saturated fatty acid chemical maps outputted from LASSO unmixing were utilized (Figure S6b, Supporting Information), which could predict mixtures of fatty acids at each pixel. To calculate the unsaturation ratio, the sum of the unsaturated chemical map, generated through linear unmixing using a C12:1‐C18:1 weighted average unsaturated standard spectra, was divided by the sum of the hyperspectral saturated chemical map. For the tracking of fatty acid production and composition dynamics (Figure 4e,f; Figures S14 and S15, Supporting Information), significant fatty acid droplets were manually segmented using the fatty acid concentration map in the last time stamp. Each droplet was manually traced and segmented frame‐by‐frame in all earlier time stamps until no fatty acid was found (Movies S5 and S6, Supporting Information).

### Single‐Cell Segmentation

Segmentation of single cells within SRS images were implemented in two steps. The protein segmentation map was first sent to CellProfiler to generate an initial segmentation.^[^ ^79^ ^]^ A customized pipeline was used for the analysis, including illumination correction, background subtraction, and edge enhancements based on the Laplacian of the Gaussian. Then a custom Matlab program was used to manually correct errors in the automated segmentation analysis using the raw SRS and protein chemical maps as a guide. When SRS images were segmented, the fatty acid channel was normalized by cell area instead of the protein channel. This normalization more accurately represented single‐cell production, whereas the protein channel normalization at the microcolony level accounted for cells growing on top of each other. Since the primary source of heterogeneity in the *Ab*TE*‐FV50 strain was at the single‐cell level, the fatty acid intensity normalized to cell area metric was utilized. Alternatively, heterogeneity seen in the ‘TesA‐FV50 strain was at the microcolony level and the fatty acid intensity normalized to protein intensity was used to represent microcolony level production.

Segmentation and tracking of phase contrast images were performed using the DeLTA 2.0 pipeline.^[^ ^64^ ^]^ Segmentation errors were corrected manually prior to downstream analysis. Growth rate of single cells was calculated using the logarithmic derivative of cell length with the following formula:

(3) $$\mu_{k}=\;\frac{1}{2\Delta t}ln\frac{L_{k+1}}{L_{k-1}}$$

Where *μ* is growth rate, *k* is the current frame, Δ*t* is the time between frames, and *L* is cell length.

### Phase Contrast Imaging

Cells were imaged with a Nikon Ti‐E microscope using a 100× objective with phase contrast imaging. Images were collected every 20 min with the microscopy chamber held at 31 °C. Production strains were grown on agarose pads containing M9 minimal media as described above for SRS imaging. After 18 h of growth, the position of the tracked microcolony was recorded and the slide was moved to the SRS microscope for endpoint hyperspectral imaging.

### Stress Responsive Reporter Strain

Cells containing the stress reporter plasmid pBbE‐ibpAB‐mRFP1 were grown on agarose pads. The cells were allowed to recover on the agarose pads for 3 h at 31 °C prior to SRS exposure. After recovery, a field of view on the pad containing several microcolonies was subject to SRS scanning at various step sizes (150 nm or 230 nm) with power held at 25 mW for the Stokes laser and 15 mW for the pump laser. Red fluorescent protein (RFP) images were taken of the scanned field of view and a nearby, un‐scanned field of view every 30 min. Since the RFP was photobleached from the SRS scan, the change in RFP of each microcolony was calculated for each condition. To account for focus differences between fluorescent images at different time points, the scanned field of view was normalized to the RFP of the nearby, un‐scanned microcolonies.

### Statistical Analysis

Hyperspectral SRS images were analyzed in MATLAB (MathWorks) using custom scripts as described in analysis method sections. Non‐image results were expressed as mean plus standard deviation unless otherwise noted in the figure caption. Sample sizes (*n*) could be found in relevant figure captions. *p*‐Values were generated by two‐tailed unpaired *t*‐tests using GraphPad PRISM software.

## Conflict of Interest

The authors declare no conflict of interest.

## Author Contributions

N.T. and H.L. contributed equally to this work. N.T. and H.L. performed experiments and conducted data analysis. M.J.D., J.X.C., and W.W.W. provided overall guidance on the project. N.T. was responsible for strain construction, production quantification, and sample preparation. H.L. performed SRS imaging. J.B.L. performed pilot experiments with a production strain. O.M.O. helped with single‐cell segmentation and tracking of phase contrast imaging experiments. D.B. helped to develop the GC‐MS protocol and quantified strain production. N.T., H.L., and M.J.D. wrote the manuscript with input from J.B.L., W.W.W., and J.X.C.

## Supporting information

Supporting Information

Supplemental Movie 1

Supplemental Movie 2

Supplemental Movie 3

Supplemental Movie 4

Supplemental Movie 5

Supplemental Movie 6

## Data Availability

The data that support the findings of this study are available from the corresponding author upon reasonable request.
