# Supplementary material for: Longitudinal Single‐Cell Imaging of Engineered Strains with Stimulated Raman Scattering to Characterize Heterogeneity in Fatty Acid Production
Source: Adv Sci (Weinh). 2023 Jun 8;10(20):2206519. doi: 10.1002/advs.202206519 (PMC10369233; doi:10.1002/advs.202206519)
Supplement: Supplementary file 1 — Supporting Information [file ADVS-10-2206519-s001.pdf]

ADVANCED SCIENCE
Open Access

Supporting Information

for *Adv. Sci.*, DOI 10.1002/advs.202206519

Longitudinal Single-Cell Imaging of Engineered Strains with Stimulated Raman Scattering to Characterize Heterogeneity in Fatty Acid Production

Nathan Tague, Haonan Lin, Jean-Baptiste Lugagne, Owen M. O'Connor, Deeya Burman, Wilson W. Wong, Ji-Xin Cheng and Mary J. Dunlop**

Supporting Information

Longitudinal single-cell imaging of engineered strains with stimulated Raman scattering to characterize heterogeneity in fatty acid production

Nathan Tague[1,a,b], Haonan Lin[1,a,c], Jean-Baptiste Lugagne [a,b], Owen M. O'Connor [a,b], Deeya Burman [a], Wilson W. Wong [a,b], Ji-Xin Cheng[a,c,d], Mary J. Dunlop*[a,b]*

Supplementary Tables

Table S1. List of plasmids used in this study.

Plasmid	Origin	Overexpressed operon	Resistance	Reference
**pSS200**	pMB1	P_{trc}-*Ab*te:G17R/A165R	Amp^R	Sarria *et al.*[38]
**pBbA5c-'tesA-vhb50-8fadR**	p15a	P_{lacUV5}-'tesA-vhb50, P_{BAD}-fadR	Cm^R	Liu *et al.*[47]
**pBbA5c-vhb50-8fadR**	p15a	P_{lacUV5}-vhb50, P_{BAD}-fadR	Cm^R	This study
**pBbA5c-CpFatB1.2-M4-287**	p15a	P_{lacUV5}-*Cp*fatB1.2-M4-287	Cm^R	This study, mutant enzyme from Hernandez Lozada *et al.*[46]
**pBbA5c-'tesA-sfGFP-vhb50-8fadR**	p15a	P_{lacUV5}-'tesA-sfGFP-vhb50, P_{BAD}-fadR	Cm^R	This study
**pSS200-sfGFP**	pMB1	P_{trc}-*Ab*te:G17R/A165R-sfGFP	Amp^R	This study
**pBbE-ibpAB-k-mRFP1**	ColE1	P_{ibpAB}-mRFP1	Kan^R	This study, based on promoter from Ceroni *et al.*[55]

Table S2. List of *E. coli* strains used in this study.

Strain	Relevant genotype	Reference
**BW25113 (wild type)**	F^- Δ(araD-araB)567 ΔlacZ4787(::rrnB-3) λ^- rph-1 Δ(rhaD-rhaB)568 hsdR514	Baba *et al.*[74]
**BW25113 Δ*fadE***	*E. coli* BW25113 Δ*fadE*, cured from Keio collection	Baba *et al.*[74]
**MG1655**	F^-, λ^-, rph-1	Blattner *et al.*[80]

Ab*TE	*E. coli* MG1655; pSS200	Sarria *et al.*[38]
**‘TesA-FV50**	*E. coli* BW25113 Δ*fadE*; pBbA5c-‘tesA-vhb50-8fadR	Liu *et al.*[47]
***Ab*TE*-FV50**	*E. coli* MG1655; pBbA5c-vhb50-8fadR, pSS200	This study
Cp*FatB1	*E. coli* MG1655; pBbA5c-CpfatB1.2-M4-287	This study
**‘TesA-FV50-sfGFP**	*E. coli* BW25113 Δ*fadE*; pBbA5c-‘tesA-sfGFP-vhb50-8fadR	This study
***Ab*TE*-sfGFP-FV50**	*E. coli* MG1655; pBbA5c-vhb50-8fadR, pSS200-sfGFP	This study

Supplementary Figures

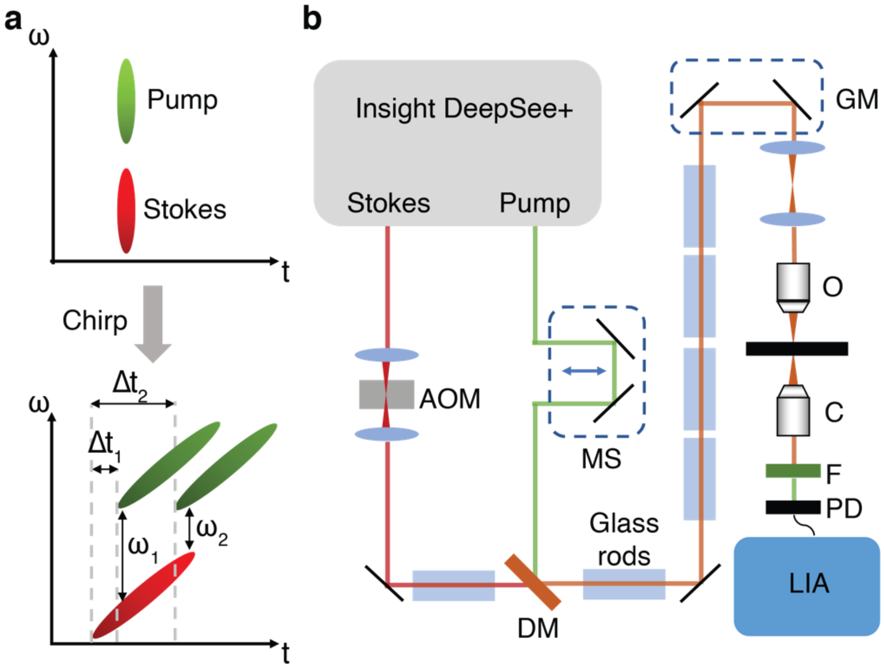

Figure S1. Hyperspectral SRS setup. **(a)** Concept of hyperspectral SRS using spectral focusing. The pump and Stokes lasers are linearly chirped by high dispersion glass rods to temporally separate the spectral components. Each temporal delay between the two pulses corresponds to a Raman vibrational mode. **(b)** Optical setup. AOM, acousto-optic modulator; MS, motorized stage; DM, dichroic mirror; GM, galvo mirrors; O, objective; C, condenser; F, filter; PD, photodiode; LIA, lock-in amplifier.

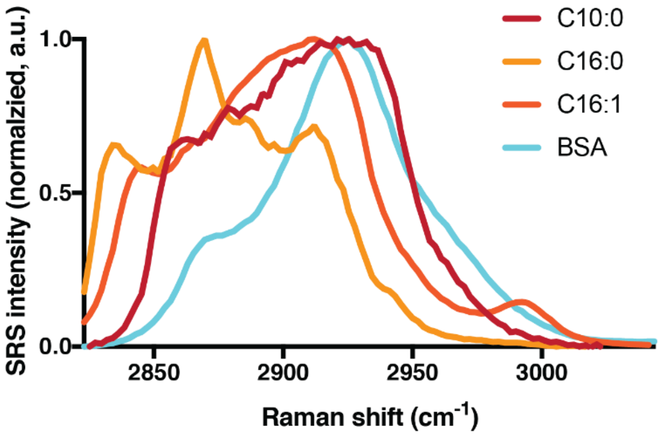

Figure S2. SRS spectra of pure standards used to analyze hyperspectral images to produce chemical maps. (BSA: bovine serum albumin, C10:0: decanoic acid, C16:0: palmitic acid, C16:1: palmitoleic acid).

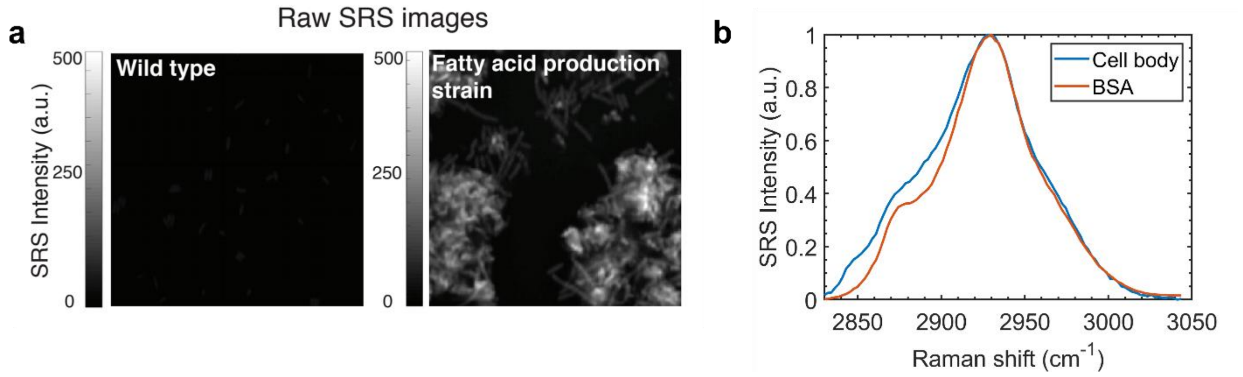

Figure S3. **(a)** Raw SRS images shown in Figure 1c of wild type and a strain overexpressing a cytosolic thioesterase (*Ab*TE*), but with both images scaled with the same color axis for direct comparison. **(b)** Comparison between the average spectrum from the wild type cells and protein reference (BSA).

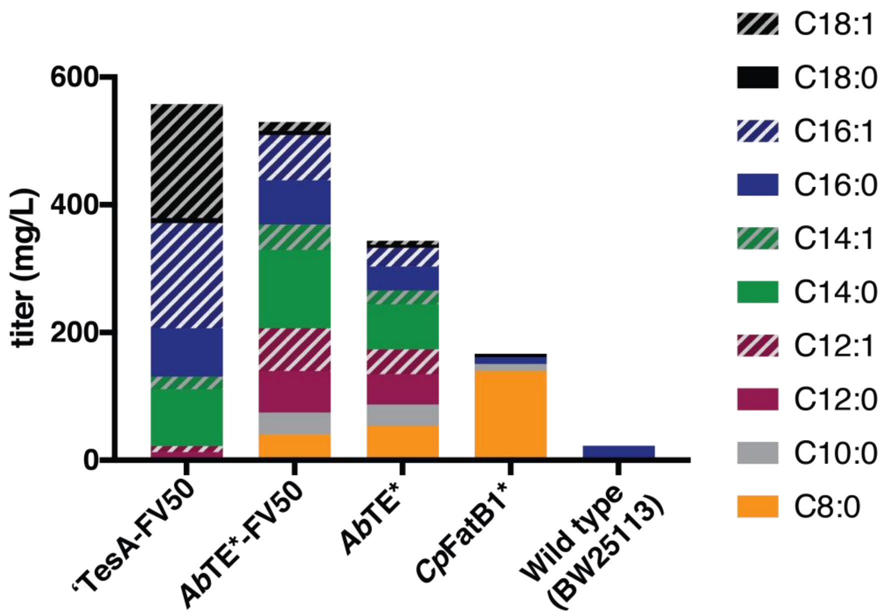

Figure S4. Fatty acid production quantification for strains in this study. GC-MS quantified fatty acid production data for each strain. Cells were grown 24 hours post thioesterase induction in liquid culture. For chain length prediction, these exact cultures were taken for SRS imaging at the same timepoint.

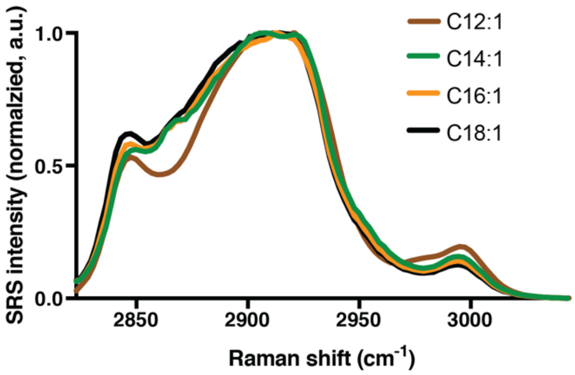

Figure S5. SRS spectra of pure unsaturated fatty acids. (C12:1: lauroleic acid, C14:1: myristoleic acid, C16:1: palmitic acid, C18:1: oleic acid).

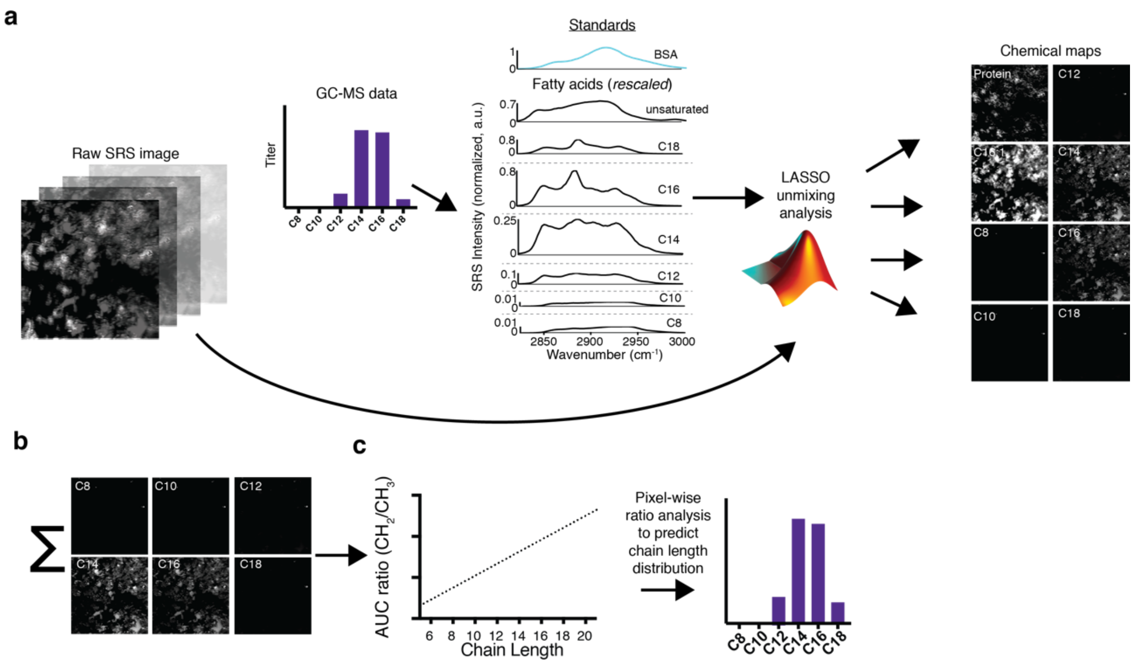

Figure S6. Analysis workflow for chain length prediction from hyperspectral SRS images. **(a)** Raw SRS images are processed with LASSO unmixing analysis. Images are unmixed using chemical standards. Fatty acid standards are rescaled according to known GC-MS fatty acid distributions and input into LASSO with a representative protein (BSA) and combined unsaturated fatty acid (C12:1-C18:1) spectra to generate chemical maps. **(b)** C8:0-C18:0 maps are summed to create a saturated chain length spectral map. **(c)** CH_2/CH_3 ratio analysis is performed on each pixel to calculate chain length and weighted by raw intensity to predict chain length distribution of the field of view.

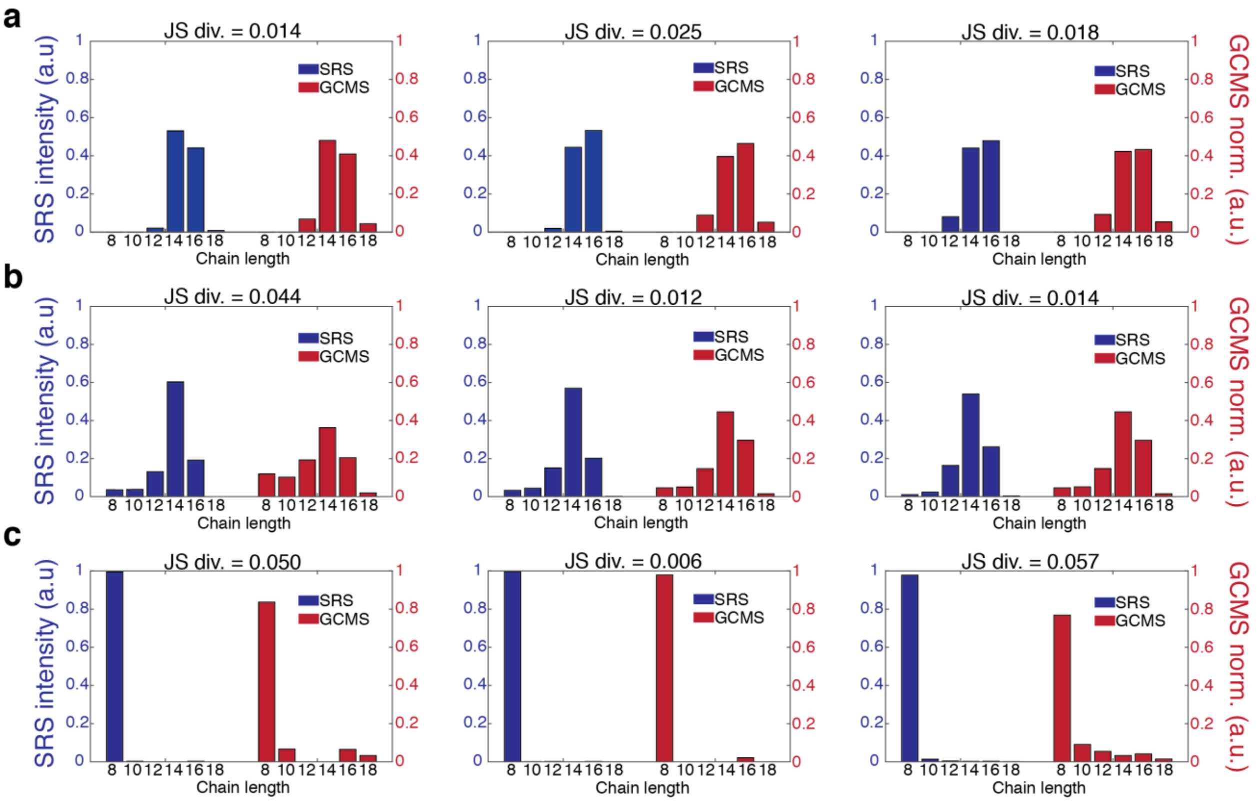

Figure S7. Quantification of similarity between SRS image and GC-MS saturated fatty acid distributions. Replicate distributions are shown with Jensen-Shannon divergence values (JS div.) for **(a)** ‘TesA-FV50, **(b)** *Ab*TE*-FV50 and (c) *Cp*FatB1* strains. Jensen-Shannon divergence values can range between 0 and 1, where smaller values correspond to distributions that are more similar.

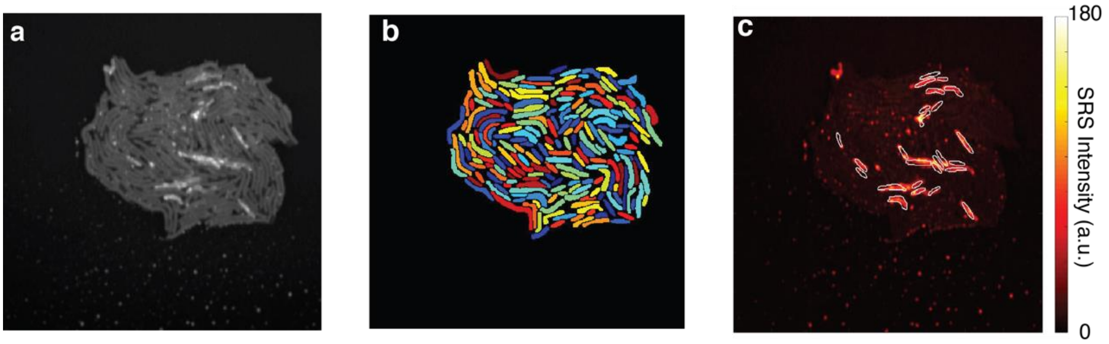

Figure S8. Single cell segmentation of a microcolony. **(a)** Raw SRS images are used to segment microcolonies to perform single cell analysis shown in Figure 3c. **(b)** Segmentation of microcolony in (a). **(c)** Segmentation of the top 25 highest producing cells overlaid on the fatty acid map of the microcolony.

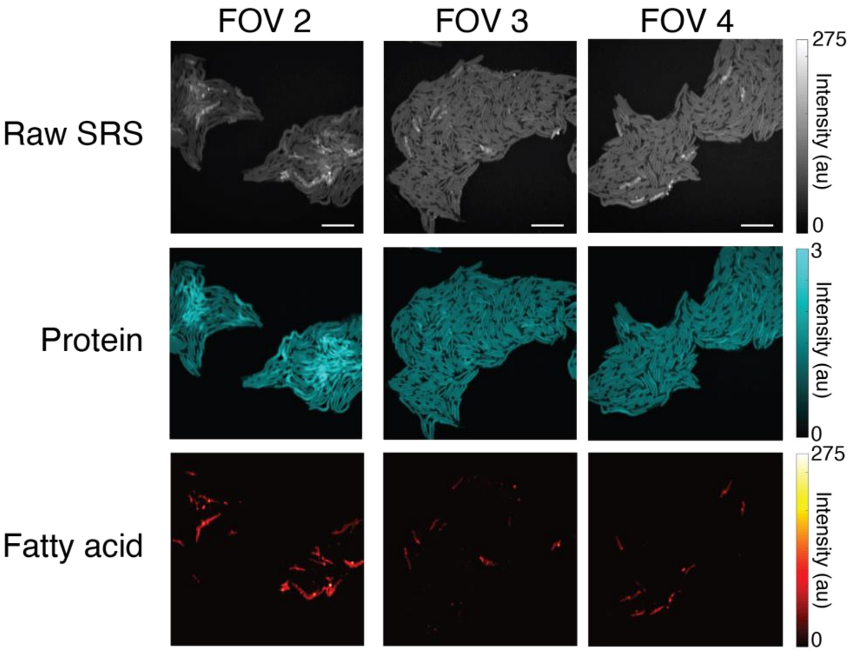

Figure S9. Intra-colony heterogeneity of the *Ab*TE* strain. **(a)** Three additional fields of view (FOV) of the *Ab*TE*-FV50 strain shown in Figure 3b. Raw SRS, protein, and fatty acid chemical maps are shown for all. Scale bars, 10 µm.

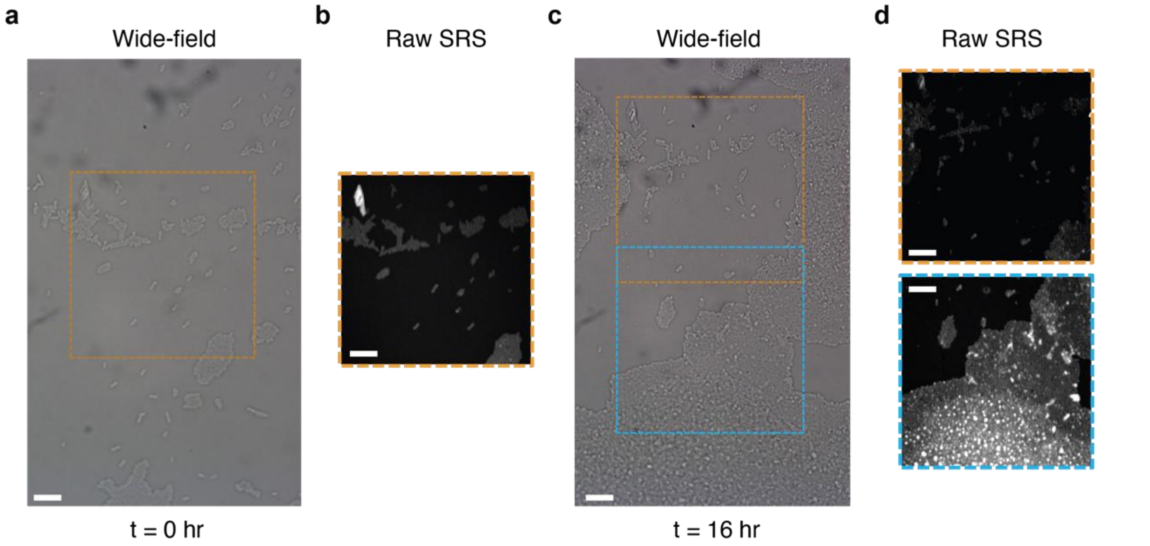

Figure S10. Testing photodamage of live *E. coli* cells. **(a)** Wide-field transmission image of *E. coli* cells at the start of the cell incubation (t = 0 hr). **(b)** Hyperspectral SRS image of the region highlighted with a yellow rectangle in (a). **(c)** Wide-field transmission image of the same field of view after incubation (t = 16 hr). **(d)** Hyperspectral SRS images of the previously scanned region (yellow rectangle in (c)) and an adjacent region without previous SRS laser exposure (blue rectangle in (c)). Scale bars, 10 µm.

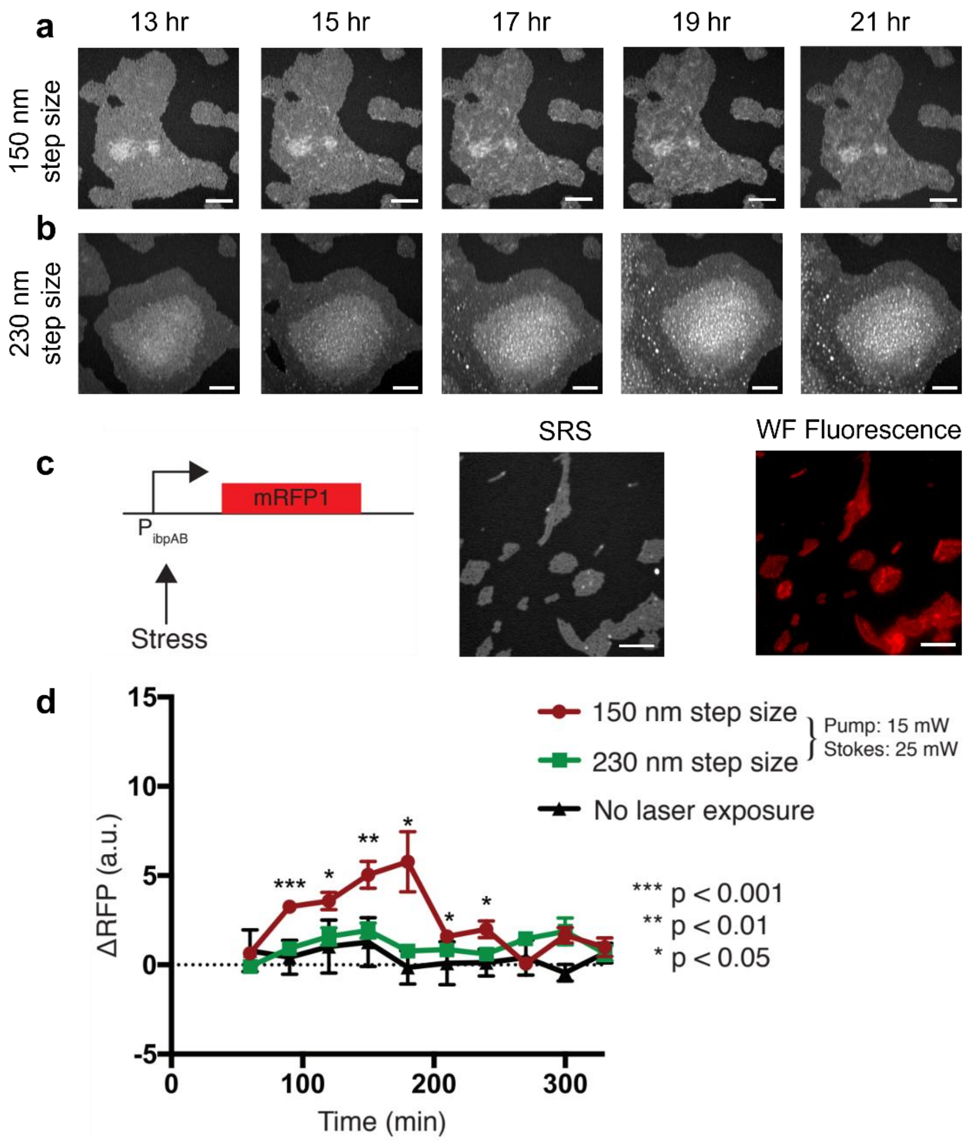

Figure S11. Optimized SRS laser powers enable live cell imaging of *E. coli*. Longitudinal SRS imaging (spectral sum of raw hyperspectral stack) of TesA-FV50 microcolonies at **(a)** 150 nm and **(b)** 230 nm step sizes. **(c)** Schematic of stress reporter, P_{ibpAB}, driving expression of mRFP1, and example of co-registered SRS and wide-field RFP fluorescence images. **(d)** Fluorescent response of cells containing the reporter after SRS exposure. Low power SRS (15mW pump and 25 mW Stokes) was tested using steps sizes of 150 nm and 230 nm. P-values compare 150 nm step size to no laser exposure (n = 9; two tailed unpaired t-test). Error bars show standard error of the mean. Scale bars, 10 µm.

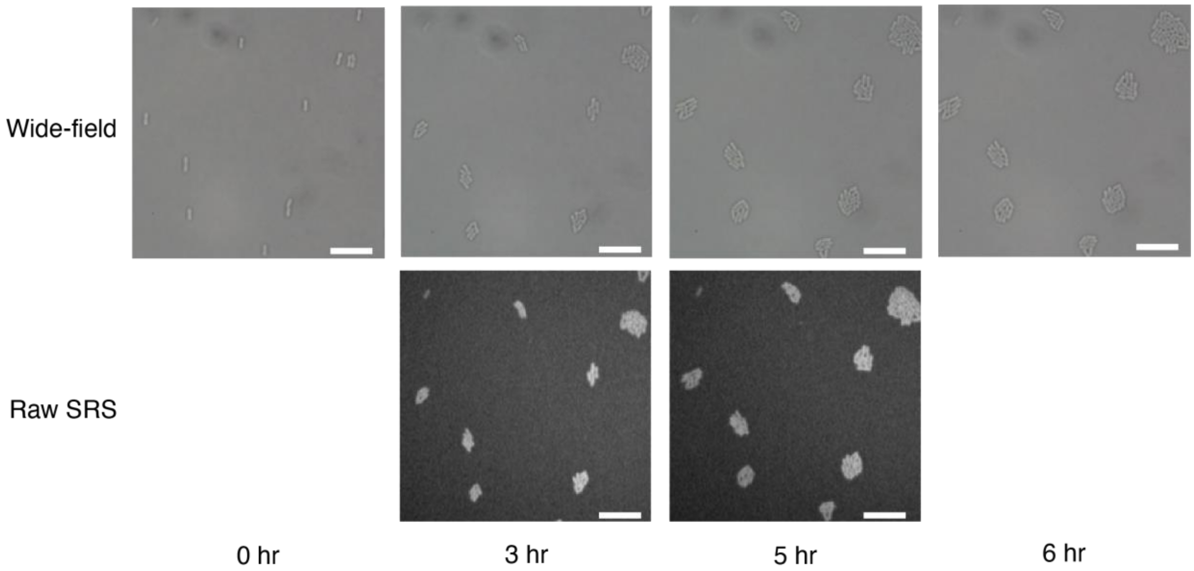

Figure S12. Live cell imaging of *E. coli* at early timepoints. Wide-field transmission image of *E. coli*, with raw hyperspectral SRS images of the same region for the t = 3 and 5 hr timepoints. Spectral summation is shown. Scale bars, 10 µm.

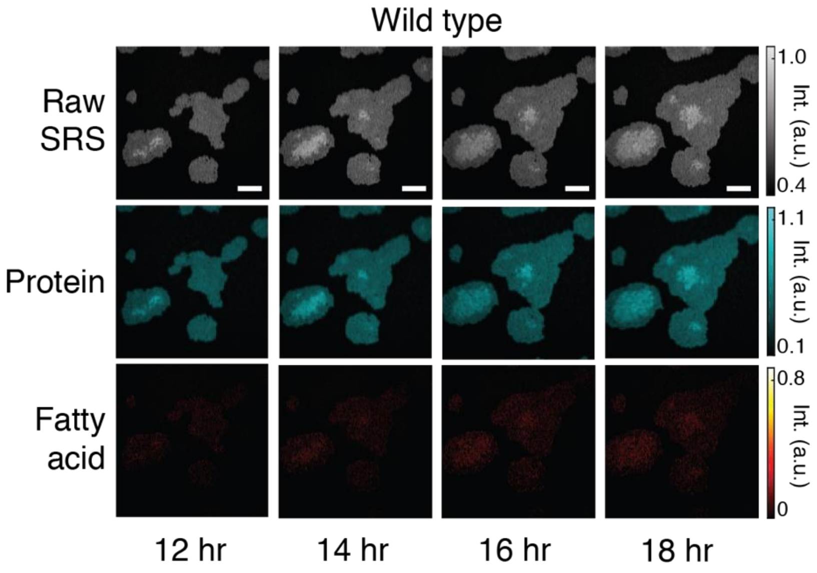

Figure S13. Time-lapse images of a wild type control strain, shown with the raw SRS images (spectral summation of the SRS image stack) and chemical maps corresponding to protein and fatty acid content.

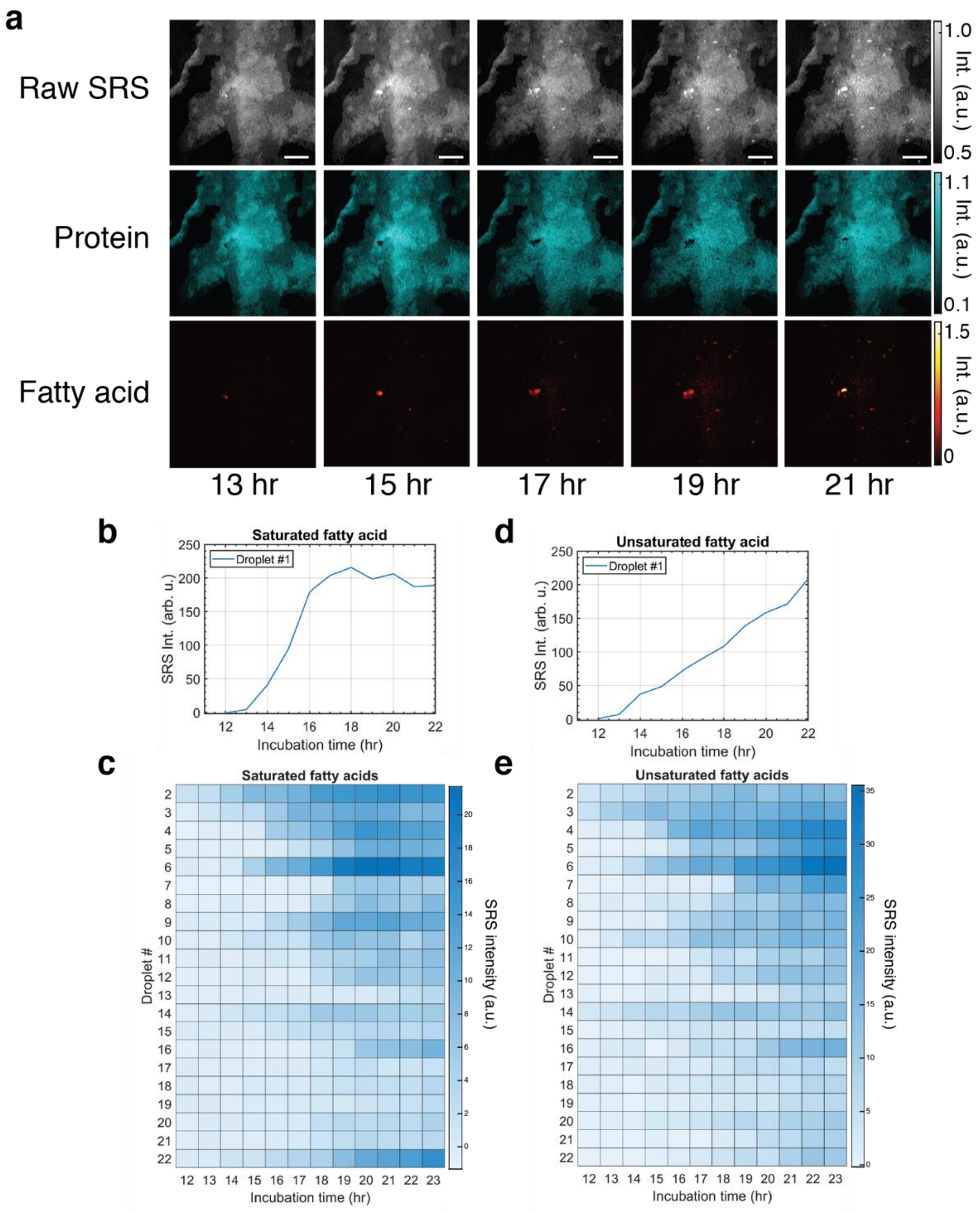

Figure S14. Time-lapse images of fatty acid production in the *Ab*TE*-FV50 strain. **(a)** Raw SRS images, protein, and fatty acid chemical maps are shown. Time values represent time grown on the agarose pad after IPTG induction. Scale bars, 10 µm. **(b-c)** Saturated content trajectories of (b) the extracellular droplet labeled as #1 in Movie S6 and (c) droplets #2-22 in Movie S6. Droplet #1 is large and has SRS intensities significantly higher than droplets #2-22, so it is plotted separately. **(d-e)** Unsaturated content trajectories of (d) extracellular droplet labeled as #1 in Movie S6 and (e) droplets #2-22 in Movie S6.

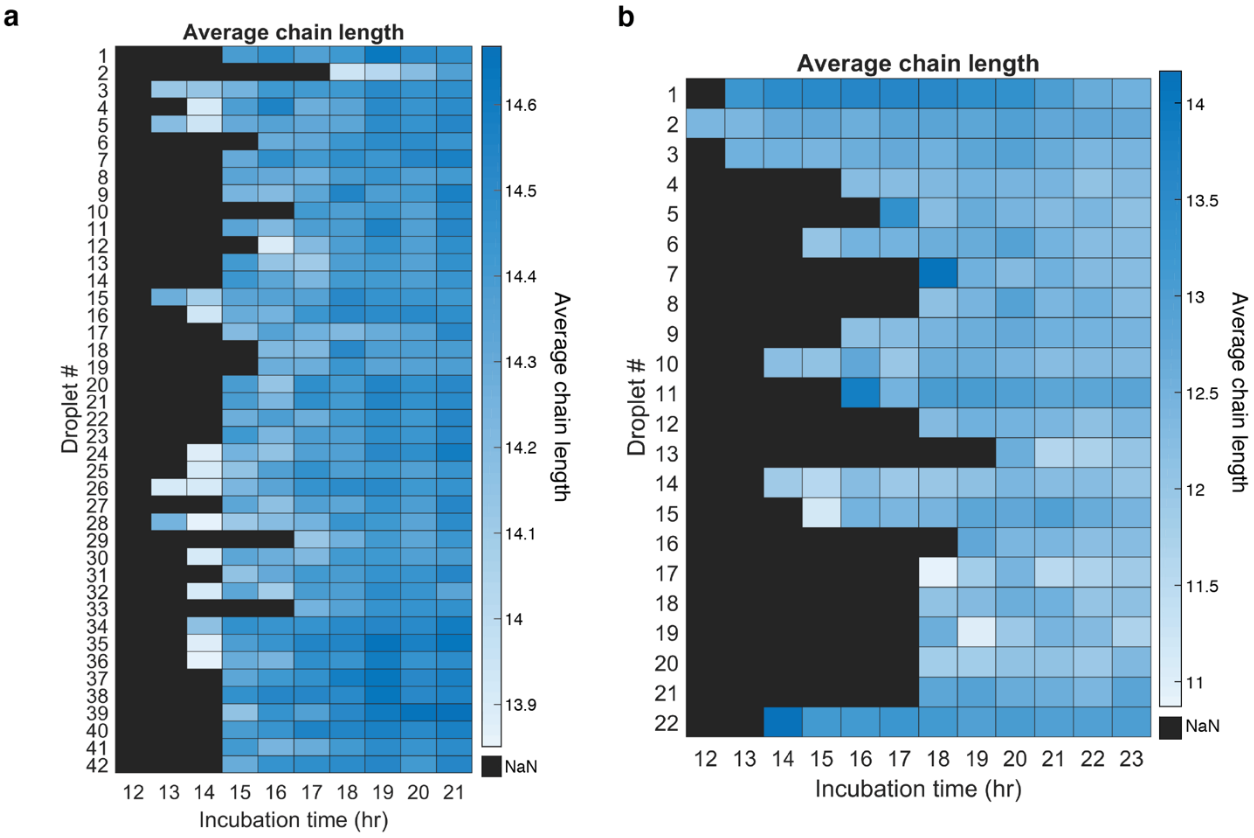

Figure S15. (a) Longitudinal chain length predictions of droplets from the ‘TesA-FV50 high microcolony from Figure 4a. **(b)** Longitudinal chain length predictions in the *Ab*TE*-FV50 microcolony from Figure S14a. NaN (black) is displayed prior to droplet formation.

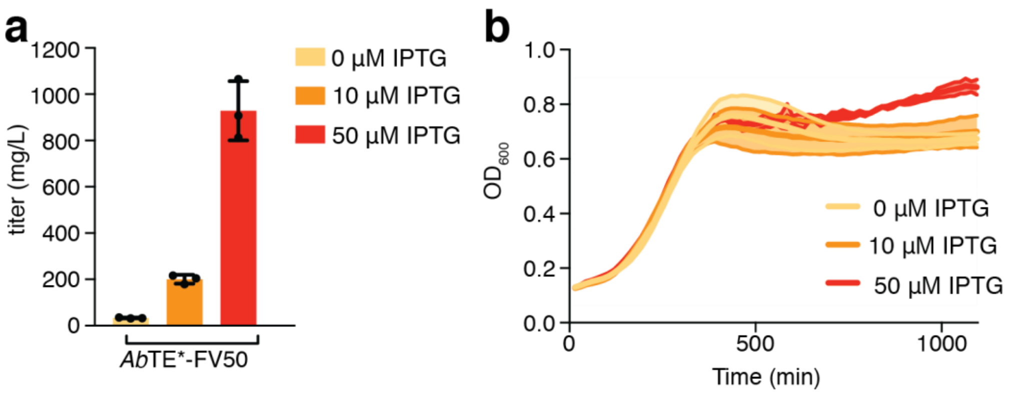

Figure S16. (a) GC-MS quantification of fatty acid production and **(b)** growth of *Ab*TE*-FV50 at varying IPTG induction levels (n = 3). Error bars, standard deviation.

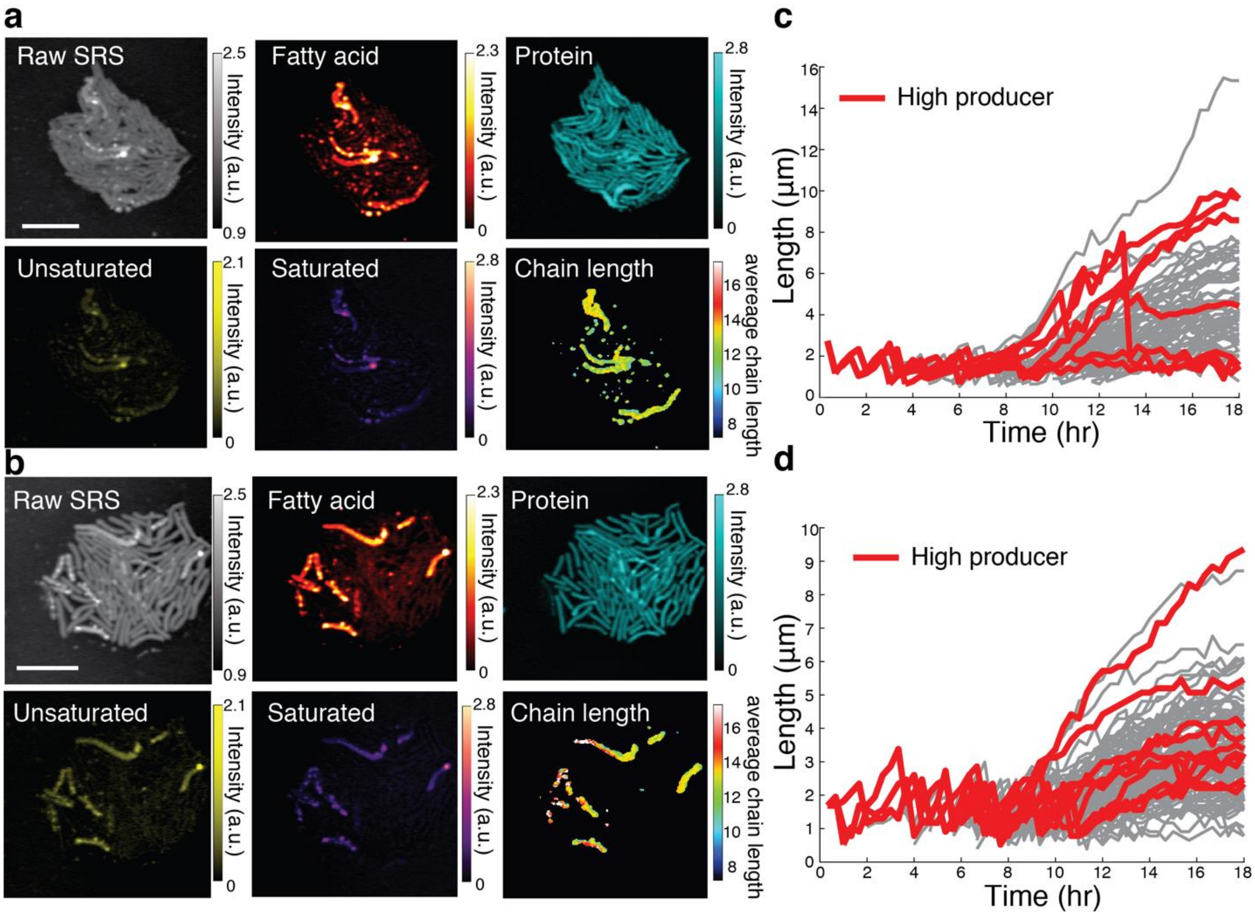

Figure S17. (a)-(b) Endpoint SRS imaging and spectral decomposition of *Ab*TE*-FV50 microcolonies tracked with time-lapse phase contrast imaging. **(c-d)** Single-cell lengths of individual cells in (a)-(b), with high producer trajectories (top 15%) highlighted in red.

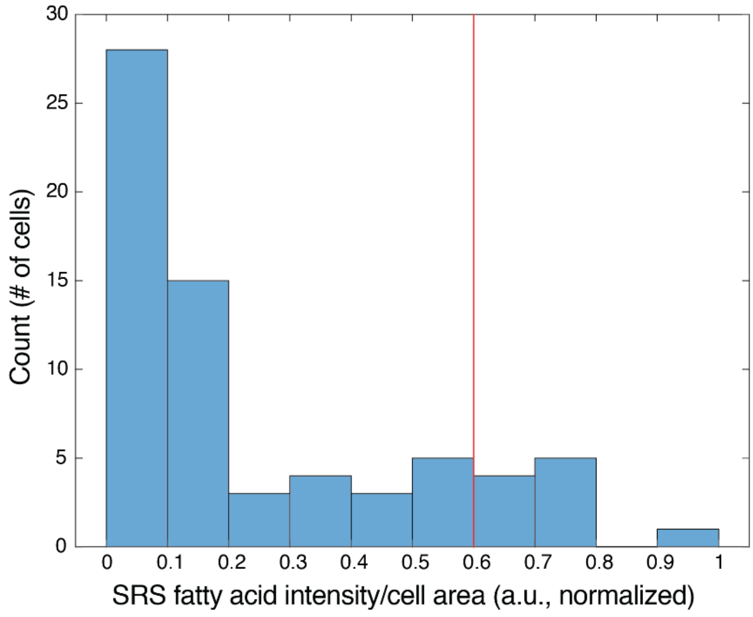

Figure S18. Endpoint fatty acid distribution of the *Ab*TE*-FV50 microcolony in Figure 5. The red line indicates the threshold we used to define high producer cells.

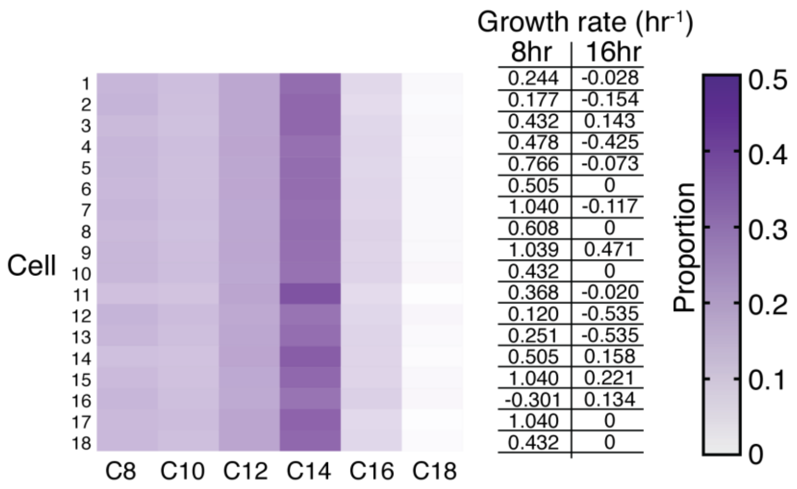

Figure S19. Saturated chain length prediction of high fatty acid cells in Figure 5, with a threshold set at 0.3 instead of 0.6. Corresponding growth rate numbers are listed. Total saturated fatty acid amount is normalized to 1.

Supplementary Movies

Movie S1. Time-lapse wide-field transmission images of the wild type strain during the live cell SRS imaging shown in Figure 3c. The white box indicates the SRS imaging region.

Movie S2. Time-lapse phase contrast images of the *Ab*TE*-FV50 microcolony from Figure 5.

Movie S3. Time-lapse phase contrast images of the *Ab*TE*-FV50 microcolony from Figure S15a.

Movie S4. Time-lapse phase contrast images of the *Ab*TE*-FV50 microcolony from Figure S15b.

Movie S5. Manually segmented droplets of the ‘TesA-FV50 strain used for compositional tracking in Figure 4e-f and Figure S15a.

Movie S6. Manually segmented droplets of the *Ab*TE*-FV50 strain used for compositional tracking in Figure S14b-e and Figure S15b.
